# Unraveling α-synuclein and amylin co-aggregation: pathological insights and biomarker development for Parkinson's disease

**DOI:** 10.7150/thno.112396

**Published:** 2025-06-20

**Authors:** Yuhang Zhou, Minchao Lai, Bowen Shu, Benguo Wang, Dian Wang, Haoran Liu, Baowan Li, Jianhe Guo, Dongjie Hu, Mingyuan Li, Cheng Zhu, Muzhi Kang, Zhong Alan Li, Renzhi Wang, Yongjuan Zhao, Rocky S. Tuan, Keying Guo, Chenzhong Li, Cheng Jiang

**Affiliations:** 1 School of Medicine, The Chinese University of Hong Kong, Shenzhen, 518172, China.; 2 Department of Neurology, The First Affiliated Hospital of Shantou University Medical College, Shantou, Guangdong, 515041, China.; 3 Dermatology Hospital, Southern Medical University, Guangzhou, Guangdong, 510091, China.; 4 Rehabilitation Department of the Second Affiliated Hospital, School of Medicine, The Chinese University of Hong Kong, Shenzhen & Longgang District People's Hospital of Shenzhen, Shenzhen, Guangdong, 518172, China.; 5 Department of Forensic Medicine, Shantou University Medical College (SUMC), Shantou, Guangdong, 515041, China.; 6 School of Science and Engineering, The Chinese University of Hong Kong, Shenzhen 518172, China.; 7 School of Instrumentation Science and Engineering, Harbin Institute of Technology, Harbin, Heilongjiang, 150001, China.; 8 Guangdong Provincial Key Laboratory of Sensing Technology and Biomedical Instrument, School of Biomedical Engineering, Sun Yat-Sen University, Shenzhen Campus of Sun Yat-Sen University, Shenzhen, 518107, China.; 9 Guangdong Provincial Key Laboratory of Materials and Technologies for Energy Conversion (MATEC), Guangdong Technion-Israel Institute of Technology, Shantou, 515063, China.; 10 Department of Materials Science and Engineering, Guangdong Technion-Israel Institute of Technology, Shantou, 515063, China.; 11 Biotechnology and Food Engineering, Guangdong Technion-Israel Institute of Technology (GTIIT), Shantou, 515063, China.; 12 Faculty of Biotechnology and Food Engineering, Technion-Israel Institute of Technology (IIT), Haifa, 3200003, Israel.; 13 Monash Institute of Pharmaceutical Sciences (MIPS), Monash University, Parkville VIC, 3052, Australia.; 14 School of Biomedical Sciences, The Chinese University of Hong Kong, Shatin, NT, 999077, Hong Kong SAR, China.; 15 Department of Biomedical Engineering, The Chinese University of Hong Kong, Shatin, NT, 999077, Hong Kong SAR, China.

## Abstract

**Background:** Patients with diabetes have a higher morbidity in Parkinson's disease (PD) than others, but the mechanism underlying this link remains controversial. The co-aggregation of α-synuclein (α-syn) and amylin has been hypothesized as a key contributor.

**Methods:** Molecular interaction analysis and co-immunoprecipitation were conducted to assess the feasibility of co-aggregation. We developed a tailored surface-based fluorescence distribution method to detect the co-aggregate in the subject's serum sample and brain-derived L1CAM-positive Extracellular Vesicles. Subjects include Health Controls (HC), PD patients and multiple system atrophy (MSA) patients.

**Results:** The co-aggregates were detected in PD patient samples, in both serum and brain-derived extracellular vesicles (EVs). We demonstrated that the co-aggregate count could distinguish PD patients from healthy individuals. Our results revealed a positive correlation between co-aggregate count and Parkinson's disease scales or diabetes markers, highlighting the role of co-aggregation in promoting PD progression. The distribution of co-aggregates demonstrated diversity among different α-synucleinopathies; a high co-aggregates count was found in EVs and serum of PD patients, but not in the serum of MSA patients.

**Conclusion:** The existence of α-syn-amylin co-aggregates was confirmed. Our findings suggest that α-syn-amylin co-aggregation may play a pivotal role in PD pathology, and have the potential as a biomarker. These results point to a potential path for early-diagnosis and therapeutic intervention.

## Introduction

The α-Synuclein (α-syn) protein is regarded as a nuisance for its aggregation-prone nature, which plays a pivotal role in a spectrum of the most intractable diseases like Parkinson's disease (PD), multiple-system atrophy (MSA), Lewy bodies dementia (LBD), etc. These diseases are often classified as “protein conformational disease” (PCD) 1-3. For long, the aggregation phenomenon has been attributed to a “seed-amplification” mechanism, where existing misfolded α-syn aggregates allure more normal proteins to join them. Conventionally it has been believed that such aggregation only occurs among homologous 7 species, as is the case with any other amyloidogenic proteins 1,2,4. However, mounting evidence implied that seeding-amplification might not be restricted to the homologous protein but may straddle different amyloidogenic proteins, which makes it a “co-aggregation” 5-8. The role of co-aggregation remains controversial, while most of work deems it to be pernicious 9-11. α-syn has been noticed to be able to interact with multiple amyloidogenic counterparts 11. The co-aggregation with α-syn was suspected to be liable to the pathogenesis and progression of α-synucleinopathies, especially PD 12,13.

We took note of a crosstalk between PD and Type 2 Diabetes Miletus (T2D). Approximately 80% of PD patients exhibit glycemic abnormities, while treatment against T2D has been shown to ameliorate or even partially reverses the PD symptom 14-17. Amylin is the symbolic amyloidogenic peptide related to T2D, meanwhile it is a frequently studied co-aggregation counterpart 18-21. Thus, we conjecture that there could be a co-aggregation going on between α-syn and amylin. Multiple works have also suggested this co-aggregation pathology in respect of molecule dynamics or cell models 22-24. It also led to another issue, that α-synucleinopathies (e.g., PD, MSA, LBD, etc.) share the identical pathogenic molecule but have distinct manifestation. Some investigations have noticed the difference in α-syn aggregate structure between α-synucleinopathies 25,26. And co-aggregation give rise to diversified aggregation dynamics, which has been remarked repetitively 8,23,27. We further deduce that it might affect outcomes of aggregation (in speed, structure, size, stability, etc.), hence the mode of pathogenesis. However, direct evidence supporting this phenomenon in patient systems remains elusive.

To acquire factual, concrete evidence from patients, we adopted the emerging concept of liquid biopsy, which considers serum biomarkers and extracellular vesicles (EV) related markers promising sources that could decipher *in vivo* activities 28-30. Investigation into the co-aggregates derived from blood, or brain-derived EVs might contribute to our understanding of the co-aggregation scenario in the brain 30-32. The value of α-syn species level (in either blood or EV) has been long treasured 33-35. In our previous work, L1CAM was found to be a selector to isolate brain-derived EV (L1EV) 28,31. We therefore hypothesized that it may also carries α-syn aggregates and α-syn-amylin co-aggregates.

Nevertheless, the available methods for their detection are still limited in scope and maturity 37-39. Current mainstream technologies for detecting protein aggregates include two classes: amplification-based Real-time quaking-induced conversion (RT-QuIC) or immunosorbent-based single molecule array (SiMoA). While these technologies have achieved high sensitivity, high sensitivity alone does not fully address this issue. To confirm a co-aggregate, the detection system must be able to quantify and output the signal that represent the two components respectively. Therefore, we adapted the idea of Surface-based Fluorescence Distribution Method, and developed our own dual-fluorescence co-localization platform on its basis 39. This system is compatible with real biofluid analytes (including raw serum and EV lysate) and its high-throughput nature enables its application in cohort-level studies. Overall, the scientific value and diagnostic potential of co-aggregate were evaluated using clinical cohort.

In this work, we studied the viability of α-synuclein-amylin co-aggregation. Specifically, we detected the presence of α-synuclein-amylin co-aggregates in liquid biopsy samples. Samples from PD, MSA, and health control (HC) groups were collected, with serum and serum-isolated L1EV lysate analyzed as the analytes. Our findings provide direct evidence for the presence of α-syn-amylin co-aggregates in clinical samples of α-syncleinopathy patients. The co-aggregate count correlated with the clinical manifestation in PD, and the distribution of co-aggregates differed between PD *vs* MSA patients.

## Results

### α-Syn and amylin form co-aggregate in vitro

**Figure 1:**
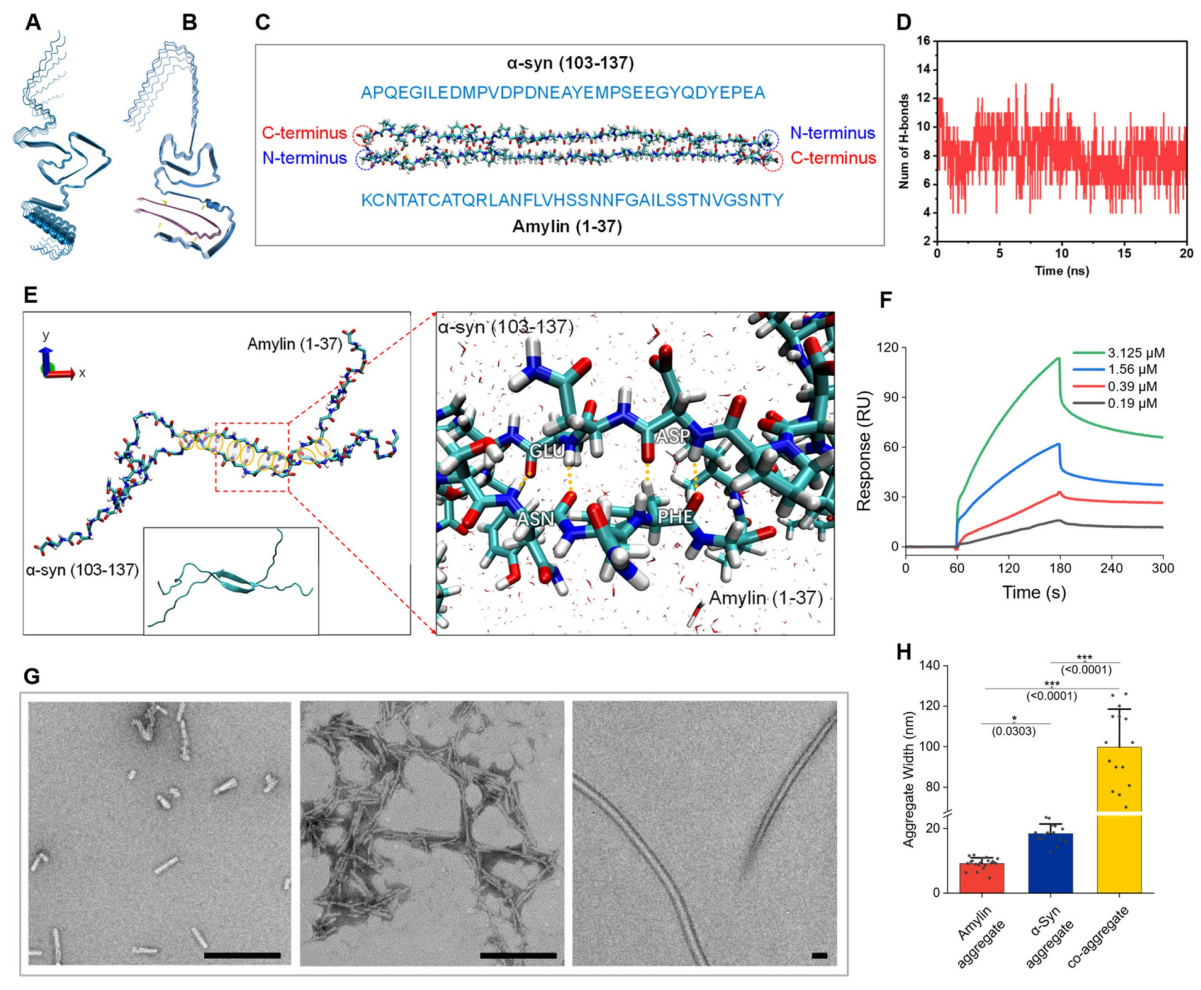
** α-Syn interacts with Amylin and form co-aggregates. A,** Predicted structure of 5-monomer aggregation of α-syn. **B,** Predicted structure of co-aggregation involving 5 α-syn and 5 amylin molecules. **C,** Initial structure of the two chains concerned. **D,** Hydrogen bond number fluctuation profile in the simulation. **E,** Final equilibrated structure at 20 ns; yellow ellipses indicate regions of hydrogen bond formation. Insets (red dashed lines) show detailed views of hydrogen bond interactions. **F,** Molecular interaction analysis of α-syn-Amylin by SPR platform. **G,** Left to right, representative TEM image of α-syn aggregates, amylin aggregate and co-aggregate, scale bar = 200nm. **H**, Comparison of aggregate widths among α-syn, amylin, and co-aggregates. Each condition was examined using three independently prepared TEM grids (technical replicates); for each grid, five random fields were analyzed. Statistical significance was calculated using one-way ANOVA with posthoc Tukey. Error bars represent mean values ± standard deviation. *p < 0.05, ***p <0.001. Source data are provided as a Source Data file.

We began our study by investigating the feasibility of the α-syn-amylin coaggregation by modeling. The Alphafold-3 was adapted to simulate the consequence of co-aggregation and provided a provisional conformation establishment. When 5 α-syn monomers aggregate, they formed stacked structure **(Figure 1A)**. While when 5 α-syn monomers co-incubated with 5 amylin monomers, α-syn and amylin formed two separate but attached stacks. The predicted structure significantly differs from pure α-syn **(Figure 1B)**. These imply that participation of amylin could affect aggregation dynamics of α-syn 25,40. We noticed that in many predicted structures, amylin tethered on the C-terminal residues of α-syn **(Figure S11)**. The negatively charged C-terminal region of αSyn is known to constitute the “fuzzy coat” of αSyn aggregates, whereas amylin is a positively charged peptide 41. Previous studies have shown that truncation or immobilization of the αSyn C-terminus accelerates its aggregation 42,43.

Therefore, molecular dynamics (MD) simulations were conducted to further investigate the a-syn-amylin coaggregation behaviors. To simplify the models, we focused on the two main liable segments with high structural confidence coefficient in Alphafold-3. Specifically, the C-terminal region of α-syn (residues 104-137) and full-length amylin were selected for the simulations **(Figure 1C)**. The initial structures of bonded α-syn (104-137) and amylin originated from the Alphafold-3 predictions. The MD simulations were carried out for 20 ns in water environment to ensure sufficient relaxation of all the molecules with pH of 7.4. It is clearly suggested that α-Syn (104-137) and amylin formed stable hydrogen bonds (H-bond) between their backbones, with an average number of 8 throughout the 20ns simulation trajectory **(Figure 1D)**. We summarized the dynamic H-bond formation in the peptide systems and listed the pairing and occupancy information of H-bond in **Figure S8A**. The occupancy of H-bond represents the ratio of formed H-bond over the total simulation time. The fragment PVDPDNEAYEM of α-syn and VHSSNNFGAILS of amylin were identified as the main residue regions forming H-bonds **(Figure S8B)**. The magnified figure highlighted the H-bond formation between amnio acids GLU-ASN and ASP-PHE (angle 35° and distance 3.0 Å, **Figure 1E**). Six amnio acid pairs formed H-bond with occupancy over 77% (MET-VAL, TYR-SER, GLU-ASN, ASP-PHE, ASP-ILE, and PRO-SER), supporting a high likelihood of a-syn-amylin coaggregation. Although many labs have repetitively conducted co-aggregation experiment, a decisive molecule-binding analysis is yet to be done 44. We conducted the binding analysis by surface plasmon resonance (SPR) method **(Figure 1F)**. A significant binding signal was detected between α-syn and amylin. The binding affinity of the two was at the μM level, which was considered a mid-range potency (ka: 5809 M^-1^s^-1^, kd: 2.136E-03 s^-1^, KD: 3.677E-07 M). The molecules used for SPR were verified to be plain monomers by Native Polyacrylamide Gel Electrophoresis (Native PAGE) and Matrix-Assisted Laser Desorption/Ionization Time-of-Flight (MALDI-TOF) mass spectrum **(Figure S9)**. It is conservative to say that it is in the same magnitude as α-syn self-aggregation 45. Recently, Hornung et al published a value of 26.7nM, which was higher than our result 46. However, their fluorescence titration method requires fluorescence labelling on the molecule, which might affect its aggregation dynamics, while our SPR method adopted native molecules. Under our SPR experiment condition, the amylin ligand was immobilized on the chip by carbodiimide crosslinker chemistry, in which steric hindrance could pose significant interference to small peptide amylin. We reasoned that the actual binding affinity of the two should be within the range of our result and Hornung's. Thus, α-syn and amylin can form a stable, or at least metastable co-aggregate.

**Figure 2:**
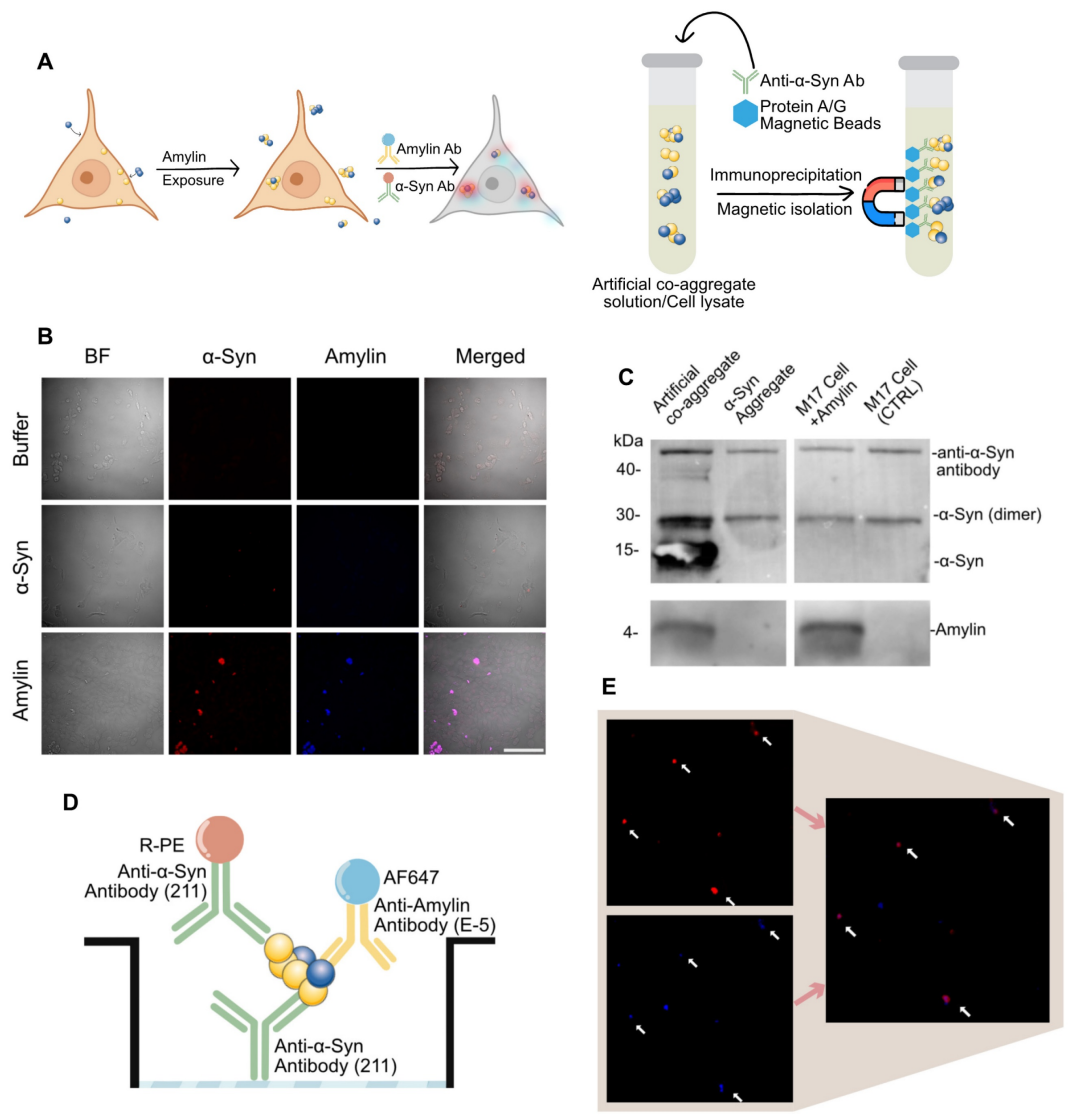
** α-Syn-Amylin co-aggregate detected by dual-antibody strategy. A,** Assay for amylin elicit α-Syn-amylin co-aggregation by M17 cell. Left, cell immunocytochemistry, right side, co-immunoprecipitation **B,** Representative two-color epifluorescence images showing Amylin exposure induced α-Syn condensation. Scale bar = 100μm **C,** α-Syn aggregates and α-Syn-amylin co-aggregates were co-immunoprecipitated using an α-Syn specific antibody (211) and then analyzed by western blotting using both antibodies. This image represents one of three independent experiments. **D-E,** The scheme of dual-channel “α-Syn-Amylin co-aggregate” detection system.

We further validated the composition of the co-aggregation product. The artificial aggregate product was pulled down by α-syn antibody, then subjected to western blot (WB). The WB bands showed that both elements were found **(Figure 2C)**. A simple characterization of aggregate structures was conducted by negative stained Transmitting Electronic Microscopy (TEM) regarding different aggregate species. We noticed that the end-point co-aggregate fibrils are morphologically different from those sole α-syn fibrils or sole amylin fibrils **(Figure 1G)**. The comparison of fiber width revealed a class of gigantic fiber in co-aggregate condition, whose width reached ~100nm **(Figure 1H)**. The aggregation condition of amylin and co-aggregation was identical, including time, temperature, mole concentration, and buffer system. Unprecedented sturdy fiber formation could be a presentation that co-aggregation occurs. Dual-labeling immunogold EM revealed that a subset of fibrils exhibited co-localization of both α-syn and amylin, indicated by the presence of both 35 nm and 10 nm gold particles along the same fibrils **(Figure S11A)**. As a control, α-syn fibrils were exclusively labeled with 35nm gold particles, suggesting heterogeneity in the composition of the co-aggregates **(Figure S11B)**. These observations support the co-aggregation.

### α-Syn-amylin co-aggregates presented in biosamples and measured by surface-based fluorescence distribution method

It was previously reported that protein aggregate species could be actively taken up by neural cells 38,47. We further reason that amylin can be taken up in neural cell culture hence elicit α-syn aggregation. Here, we conducted a transient exposure of amylin or α-syn aggregate on M17 cell line, then subjected to immunocytochemistry and pull-down analysis **(Figure 2A)**. Amylin exposure significantly induced α-syn signal compares to blank control or α-syn aggregate as shown in immunocytochemistry. The fluorescent signal of α-syn and amylin were co-localized, and the cells that presented signal showed a shriveled appearance **(Figure 2B)**. These results implied that amylin exposure exacerbates α-syn aggregate pathology, we thus reasoned that there might be a direct co-aggregation of amylin and α-syn.

To confirm the existence of co-aggregation, we conducted two pull-down experiments on both cell lysate and artificial co-aggregates. The pull-down was done by α-syn antibody (211), which aims to collect all α-syn aggregate and separate potential amylin component by electrophoresis. For artificial co-aggregates, both bands were found in one lane. As for cell lysate of above treated cell, both band of amylin and α-syn were also found in one lane **(Figure 2C)**. The phenomenon that two targets were detected in one lane demonstrated that these two were isolated collectively by α-syn antibody.

A major obstacle in detecting co-aggregates in clinical samples was that there is no statutory assay for co-aggregates. Considering previous data had shown that the abundance of aggregate species in real sample was poor, we deduced that co-aggregate species must be scarce 35. In align with previous imaging-based detection methods, we managed to establish our co-localization-based detection setting 38,48. We introduced the pigeonhole principle that using a pair of identical antibodies to rule out signal from α-syn monomers 37. In this unique sandwich configuration, the epitope on the α-syn monomer is occupied by the capture antibody, thus cannot be recognized by the detection antibody again. However, α-syn aggregates are supramolecules containing multiple identical epitopes, allowing recognition by the detection antibody, which makes it a “aggregate only” detection system **(Figure 2D)**. On the basis of which, we further introduce a fluorescent reporter antibody against amylin. Lastly, we measured the co-localized fluorescent particles and adapt it as a confirmed co-aggregate count **(Figure 2D-E)**. The artificial co-aggregates were adopted to verify the performance of the method. In both channel, massive fluorescent particles were detected, while amylin was significantly less than α-syn, this proved that our fluorophore setting does not pertain to fluorescence resonance energy transfer effect **(Figure S1B)**. When the sample was diluted by 5× for seven steps, a gradient decrease in particle counting in both channels was observed **(Figure S1C-D)**. The data processing was accomplished by automated algorithms, and the detailed quantification code was given in “Methods” section.

### Evaluation of co-aggregate count in serum and brain-derived extracellular vesicles

To evaluate the role of co-aggregation in PD pathology, we compared its occurrence in patients and healthy individuals. Using liquid biopsy, we investigated its distribution in EV subpopulations to clarify their role at the lesion site. Additionally, the diagnostic potential of co-aggregate was evaluated by the receiver operating characteristic (ROC) curve method. In this work, co-aggregates demonstrated superiority than α-syn self-aggregate biomarker.

**Figure 3:**
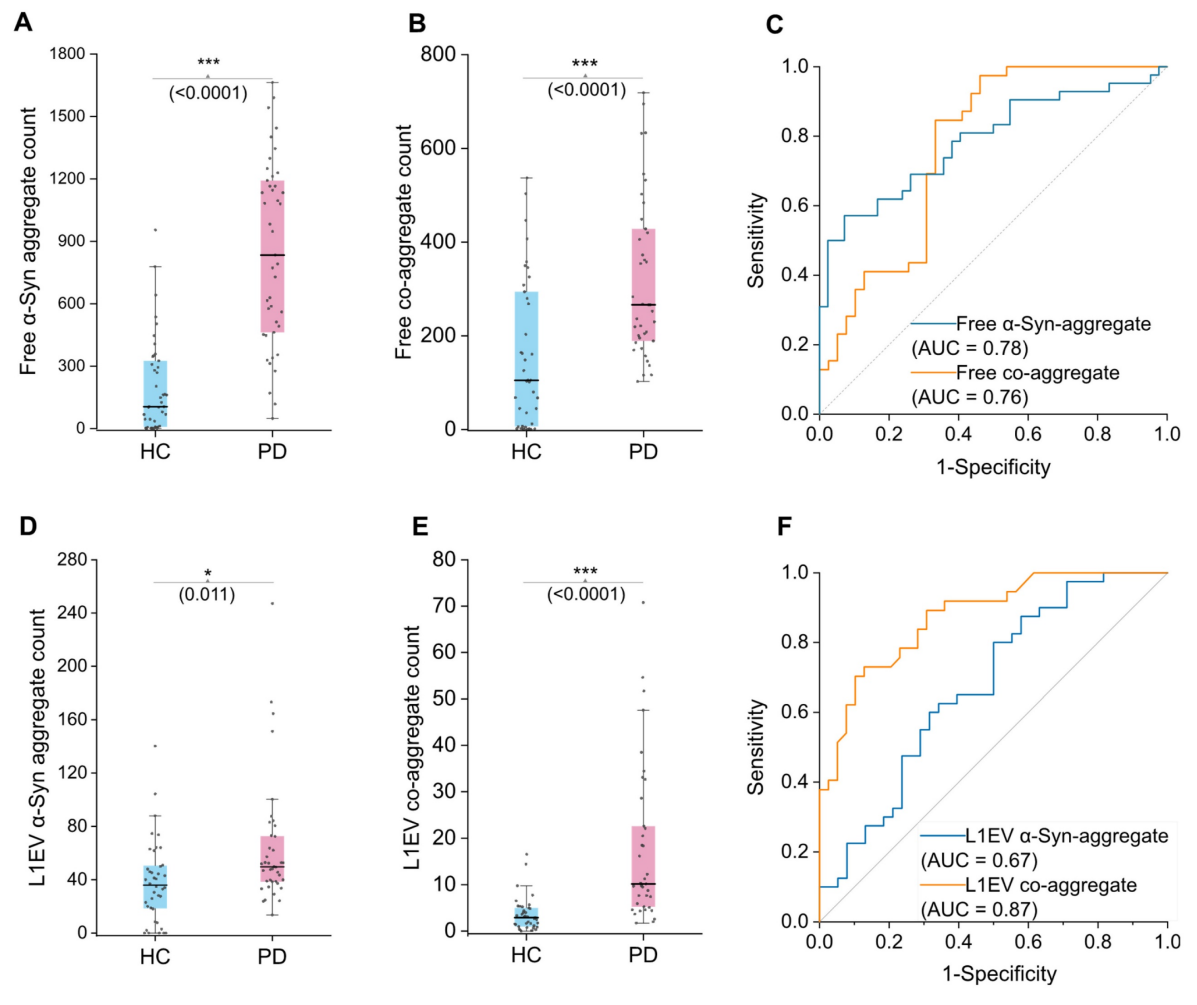
** Diagnostic value of co-aggregate count in separating HC and PD group using EV and serum sample. A,** α-syn aggregate comparison between HC and PD group in serum samples (N = 21 participants; each sample was measured in duplicate, with ~6 readout points per replicate)**. B,** Co-aggregate count comparison between HC and PD group in serum samples (N = 21, duplicated measurements as above)**. C,** Performance that serum free floating α-syn or co-aggregates distinguish PD from HC was assessed by AUC. **D,** α-syn aggregate comparison between HC and PD group in L1EV sample lysates (N = 20, duplicated measurements as above)**. E,** Co-aggregate count comparison between HC and PD group in L1EV sample lysates (N = 20, duplicated measurements as above)**. F,** Performance that L1EV α-syn or co-aggregates distinguish PD from HC was assessed by AUC. Statistical significance was calculated using an unpaired two-sample t-test. *p < 0.05, **p < 0.01, ***p < 0.001*,* ns, non-significant (*p* ≥ 0.05). Error bars represent mean ± standard deviation. Source data are provided as a Source Data file. AUC, Area under curve. Free, refers to aggregate species in “free floating state”.

We analyzed serum samples from PD patients (n = 21) and health controls (HC, n = 21). Target protein aggregates in free floating state were measured first, by directly using diluted serum as analyte (hereinafter referred to as "free" count). α-Syn aggregate level is a recognized biomarker of PD in various analytes, and herein we adopted it as a reference marker 39,49. Both α-syn aggregate and co-aggregate readouts were found elevated in PD compared to HC **(Figure 3A)**. The co-aggregate count performed similarly to α-syn aggregate, with area under the ROC curve (AUC) value of 0.76 *vs* 0.78 respectively. (95% CI: 0.69-0.88 and 0.66-0.87 respectively) The performance of co-aggregates did not significantly surpass α-syn aggregate. Therefore, these results suggested that excess α-syn in peripheral circulation (potentially from sources like red blood cells) may have masked α-syn and co-aggregate count in the serum free floating components.

To address this issue and improve specificity conceptually, we further analyzed brain-derived EVs. Brain-derived EVs are featured by surface L1CAM molecule (L1EVs), and the isolation was done using a magnetic separation protocol, as we previously reported 50. Samples from PD patients (n = 20) and health controls (HC, n = 20) were used. (hereinafter referred to as "L1EV" count) For each sample, we measured both the co-aggregate level as well as α-syn aggregate level. Previously reported that α-syn level of L1EV could distinguish PD patients from health controls better than serum free floating α-syn 33. We therefore surmised that L1EV carried α-syn aggregate and co-aggregate may also perform better than free floating ones. In our samples, the α-syn aggregate levels in L1EVs were indeed able to distinguish PD from HC, but with rather poor sensitivity **(Figure 3D)**. The AUC value of α-syn aggregate count was merely 0.67 (95% CI: 0.55-0.79) **(Figure 3F)**. We then measured the co-aggregate levels, and the result revealed a significant difference between PD and HC groups **(Figure 3E)**. Notably, the sensitivity of the analysis was markedly improved to 0.87 (95% CI: 0.79-0.94)** (Figure 3F)**. Compared to the α-syn aggregates, co-aggregates demonstrated superior sensitivity and reduced overlap. This highlighted the unique advantage of L1EVs carried co-aggregates in detecting PD, meanwhile a testament that α-syn-amylin co-aggregates exist in the circulation system of PD patients.

**Figure 4:**
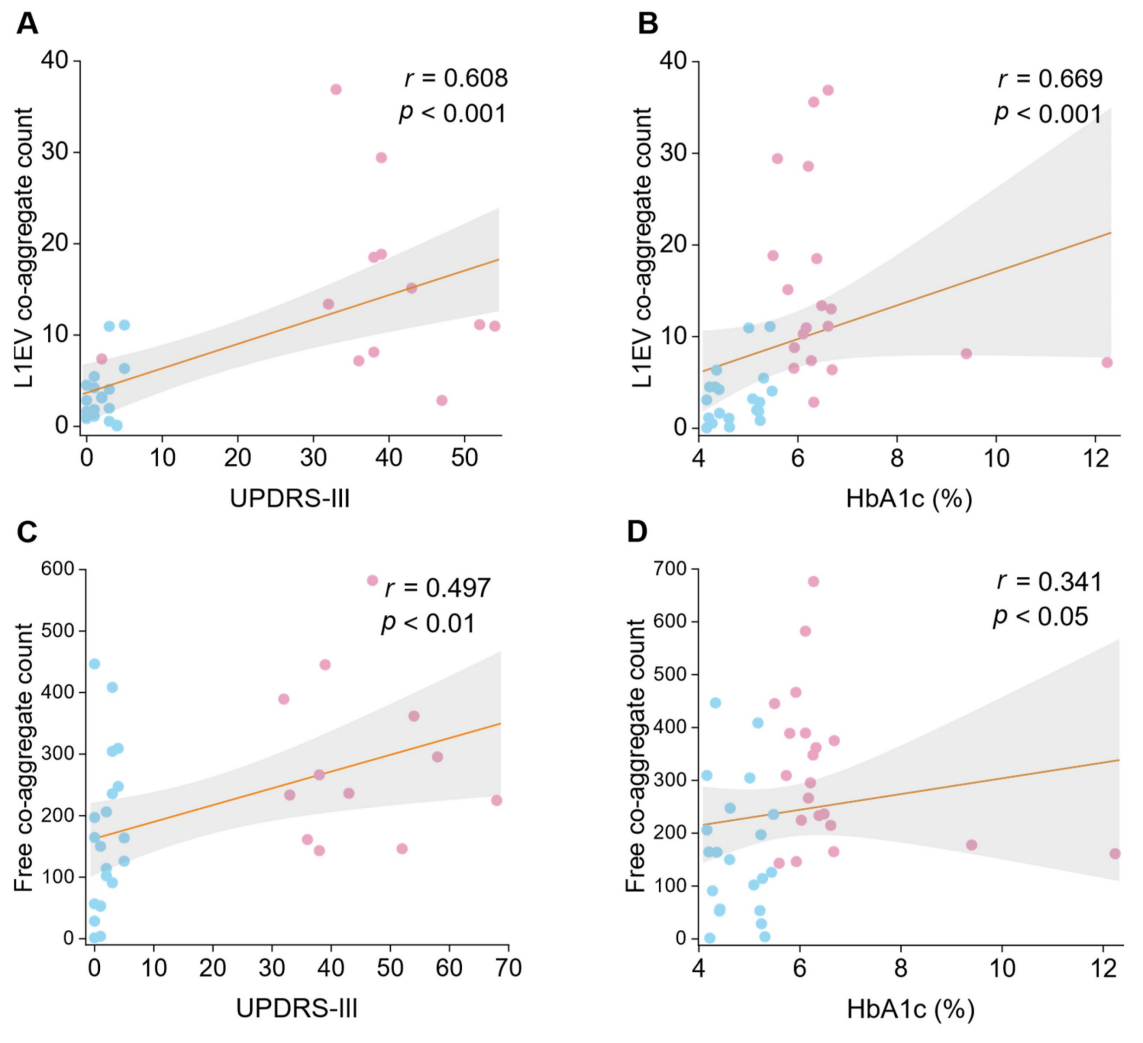
** Correlation analysis between co-aggregate counts and diabetic markers or PD scales.** Dotted lines represent Spearman rank correlations (with r and p values as indicated). Pink dots represent the PD group, while blue dots represent the HC group. **A,** Spearman correlation evaluation of L1EV co-aggregate counts with UPDRS-III score (N = 21 participants; each sample measured in duplicate; data points represent the average of ~6 measurements per replicate). **B,** Spearman correlation evaluation of L1EV co-aggregate counts with HbA1c percentage (N = 20). **C,** Spearman correlation evaluation of serum sample's co-aggregate counts with UPDRS-III score (N = 21). **D,** Spearman correlation evaluation of serum sample's co-aggregate counts with HbA1c percentage (N = 21). Source data are provided as a Source Data file.

We further hypothesized that if the linkage between PD and diabetes pathology lies on the co-aggregation, if so, a correlation between their respect indicators might be observed. Hemoglobin A1c (HbA1c) percentage was considered the most classic indicator of diabetic status. In serum samples, free floating co-aggregate count showed a weak partial correlation with HbA1c (**Figure 4A**; *r* = 0.314, *p* < 0.05). However, a stronger correlation was identified in L1EV samples (**Figure 4C**; *r* = 0.561, *p* < 0.001). These results suggest that elevated HbA1c percentage is associated with higher co-aggregate count, particularly in brain-derived L1EVs (Spearman's rank correlation coefficient *r* = 0.341 vs *r* = 0.561). Fasting plasma glucose (FPG) level was also adapted for correlation analysis, but no significant correlation was observed, regardless in plasma or L1EV (**Figure S6F-L**). These findings supported that diabetic protein aggregation could facilitate PD pathogenesis by promoting co-aggregate formation.

Currently, symptomatology-based scales remain the primary diagnosis criteria for PD. In our analysis, serum co-aggregate count showed no significant correlation with Unified Parkinson's Disease Rating Scale Part III (UPDRS-III) score (**Figure 4B**; *r* = 0.128, *p* = 0.707). In contrast, co-aggregate counts in L1EV sample exhibited a significant positive correlation with UPDRS score (**Figure 4D**; *r* = 0.582, *p* < 0.01). These results indicated that co-aggregate may contribute significantly to the progression of PD.

Non-motor symptoms were measured by several other scales. Cognitive function was measured by Montreal Cognitive Assessment (MoCA), mental state was measured by Mini-Mental State Examination (MMSE) scale, and the ability of self-care was measured by scale of Activities of Daily Living (ADL). The extent and anxiety were evaluated by Hamilton Anxiety Rating Scale (HAMA) and Hamilton Depression Rating Scale (HAMD), respectively. Scales mentioned herein are only used on PD patients. In serum samples, co-aggregate count inclined to be negatively correlated to MoCA and MMSE, positively correlated to HAMA and HAMD, and non-correlated with ADL, but these correlations were not significant **(Figure S6A-E)**. In L1EV measurements, co-aggregate count presented an inclination that it was positively correlated to MoCA and MMSE, negatively correlated to ADL, non-correlation with HAMA and HAMD, but these were still non-significant **(Figure S6G-K)**. Surprisingly we found that the trend of MoCA and MMSE correlating with co-aggregate count was opposite regarding their source (serum or L1EV). It might indicate the two different trafficking pathways of the co-aggregate but a larger sample number is needed for validation to clarify these findings in the future.

### Co-aggregate distribution differs in differing α-synucleinopathies

Recent studies have highlighted significant structural differences in α-syn aggregates among α-synucleinopathies, such as PD and MSA 26. We hypothesized that such difference could result from potential engagement of co-aggregation mechanism. To investigate this possibility, we conducted a small cohort study, which comprised three groups: HC, MSA patients, and PD patients. Both serum and L1EV samples were analyzed to evaluate α-syn and co-aggregate levels across the groups.

**Figure 5:**
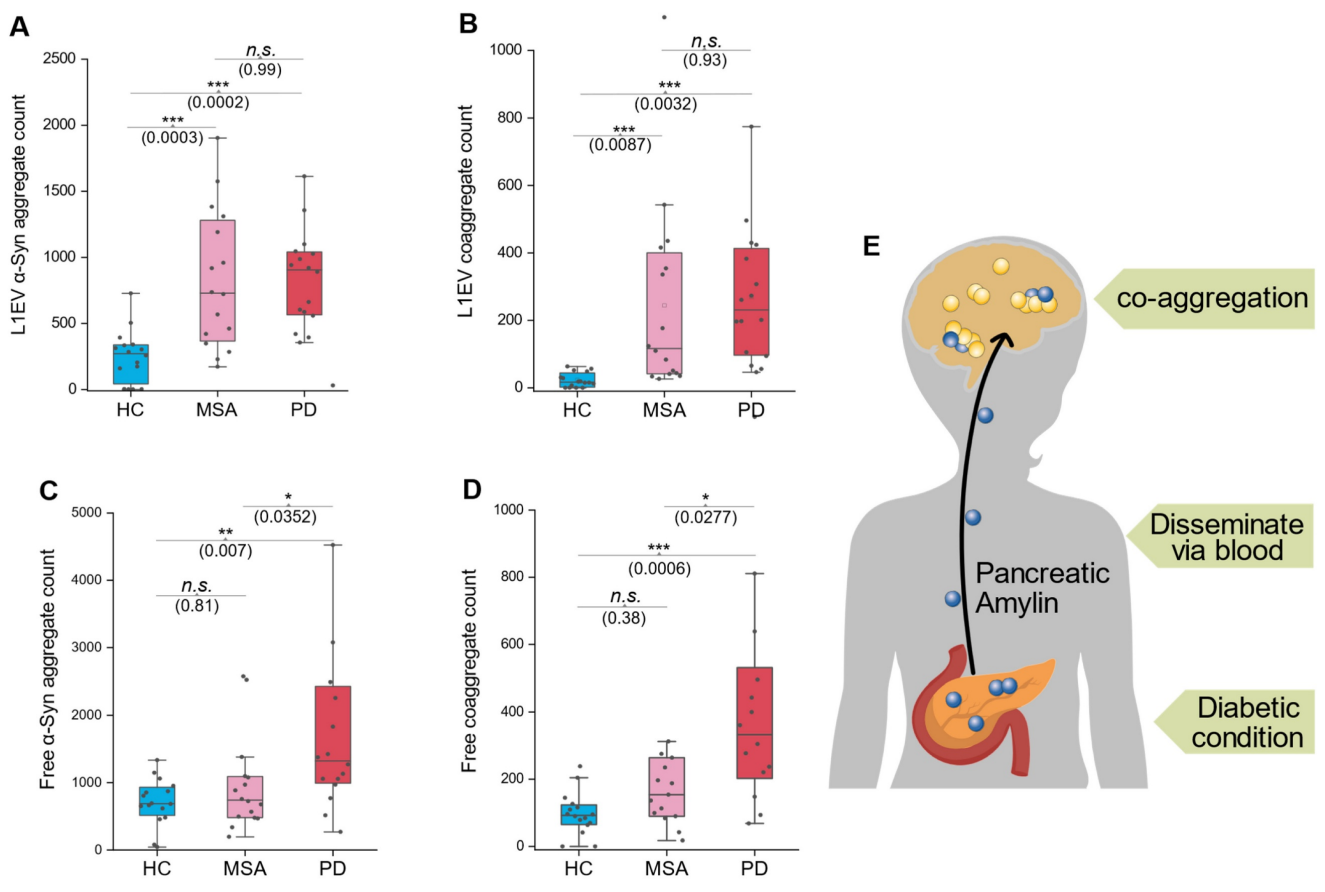
** Co-aggregates distribute distinctively in different α-synucleinopathies. A,** α-syn count comparison in L1CAM-EV lysates. **B,** Comparison of co-aggregate counts in L1CAM-EV lysates. **C,** Comparison of free-floating α-syn counts in serum samples. **D,** Comparison of free-floating co-aggregate counts in serum samples. **E**, Schematic diagram shows the hypothesis that pancreatic amylin spread to the brain via blood and triggers coaggregation with α-syn. N = 8 participants per group; each sample was measured in duplicate. Statistical significance was calculated using one-way ANOVA with posthoc Tukey. Error bars represent mean values ± standard deviation. *p < 0.05, **p < 0.01, ***p < 0.001*,* ns, non-significant (*p* ≥ 0.05). Source data are provided as a Source Data file.

In L1EV samples, both α-syn and co-aggregate count significantly elevated in both MSA and PD group compared to HC group **(Figure 5A-B)**. However, in serum samples, elevated levels of α-syn and co-aggregates (free-floating non-EV markers) were observed exclusively in the PD group, with no significant changes in the MSA group **(Figure 5C-D)**. These findings regarding serum free floating aggregates align with our earlier observations regarding serum/L1EV aggregate elevation in PD, supporting the idea that pancreas-released massive amylin disseminate into the brain and triggers co-aggregation **(Figure 5E)**. Specifically, co-aggregates in MSA cases were elevated only in L1EV-derived samples but not in serum non-EV format, highlighting a potential divergence in the aggregation pathways between MSA and PD. In fact, these results were quite striking, as we did not expect the amylin signal to be detected in MSA patients. Therefore, we further hypothesized that there were small amount of amylin carried exclusively by EVs, but amount of which was minimum compares to those in PD. This observation raises intriguing questions about the origin of amylin components within brain-derived EVs and its potential role in α-syn co-aggregation. We concede that these results need to be confirmed by larger cohorts.

These results suggest a potential diagnostic approach to distinguish MSA from PD. By comparing co-aggregate or α-syn aggregate levels between serum and L1EV samples, we propose the following criterion: if the elevation is synchronized in both serum and L1EV, the patient is more likely to have PD. Conversely, if the elevation is observed exclusively in L1EV-derived samples, the patient is more likely to have MSA. This approach underscores the diagnostic value of source-specific aggregate analysis and warrants further investigation in larger cohorts.

## Discussion

Growing evidence implies that our previous understanding regarding protein aggregation was incomplete, and the importance of co-aggregation has been long neglected 51,52. Previous self-aggregation hypothesis cannot explain the fact that PCDs tend to have statistical and prognostic linkage 53. Based on the co-aggregation hypothesis, direct linkage between different PCDs has been reiterated but there is still no direct proof 19,54,55. In this work, we successfully detected co-aggregate in PD serum samples. Co-aggregate count in samples from PD patients was significantly higher than those from healthy individuals, in both free-floating and L1EV cargo format. Moreover, co-aggregate count showed a positive correlation with disease progression in PD group. We believe that this can be regarded as the direct evidence that co-aggregation does play a crucial role in PD pathogenesis. Compared to postmortem samples, our results are more convincing since they ruled out unnatural co-aggregation induced by tissue processing, especially for frozen brain samples, offering high transitional value for future non-invasive clinical practice.

The value of co-aggregate is not restricted to deepening our understanding of PD pathology, it also has the potential to serve as a biomarker. Currently, α-syn-based biomarkers is considered the most promising candidate in clinical setting 56,57. We compared α-syn aggregate signal and co-aggregate signal in our platform, and found that the performance of co-aggregates was significantly superior. Correlation analysis also showed that the co-aggregate count is highly correlated to disease progression. In particular, L1EV-based analyte demonstrated better results than serum free floating ones, indicating neuron derived EV may reflect more accurate molecular pathology compared to free-floating species due to its higher brain tissue specificity. The distribution of co-aggregates (both free floating in serum and in L1EVs) was different in PD and MSA. That is to say, by comparing the presence of co-aggregate in L1EV and free-floating ones, we may tell apart PD and MSA. This discrepancy led to a hypothesis that the pathogenesis of PD may be a consequence of amylin transported from the pancreas, while MSA appears to be an indigenous α-synucleinopathy in the brain. Moreover, it also implied to us that it might be pragmatic to screen potential PD patients out of the diabetic population to achieve an ultra-early diagnosis. It suggested a new paradigm that a common PCD can be seen as potential neurodegenerative disease diagnosis pool, in other words, potential co-aggregates are of high translational value for clinical practice in a non-invasive way.

Our artificial fibrillation experiment showed very different outcomes regarding self-aggregation or co-aggregation, implying intrinsic structure difference. Recent reports have found that significant structural differences emerge when using different sources of seeds to perform α-syn artificial aggregation. The seeds from PD or MSA patients turned out to induce diverse aggregate formations 26,58. Our findings support the idea that this might result in co-aggregate formation. It also helps to explain the morphological differences observed in various co-aggregation studies 23,59. Taken together, co-aggregation might contribute to many PCDs to a greater extent than we know. These results urge us to revisit those pathological aggregates and plaques, especially focusing on their composition and structure. A detailed kinetic model of co-aggregation should be developed.

The phenomenon that amylin boosts aggregation dynamics of α-syn has been endorsed by various labs 22,24,46. Many clues have shown that amylin aggregates possess a strong ability for transmembrane travel, either via blood or carried by EVs 24,60-62. In general, we can infer that the amylin from pancreas might be transferred to brain and trigger co-aggregation together with α-syn, and vice versa 23,24. Similar cases for other amyloidogenic protein combinations have been found, thus we can extend this conclusion that many PCDs might be interlocked 11-13,59. We conjecture that diabetic conditions might be a common trigger many neurodegenerative diseases have epidemiological connection with diabetes. Furthermore, the “co-aggregation combinations” that might exist shall be extensively screened and studied.

Over the years, α-syn-amylin co-aggregation has been hunted down incessantly. Horvath et al found the first evidence that α-syn-amylin presented a synergetic aggregate-promoting dynamics 22. Later on, more evidences were found in animal models and post-mortem specimen 24,47. To date, the focus of debate is whether such co-aggregation really occurs in patient's system. Our work has presented the most direct evidence that co-aggregate does exist, and it does correlate with PD progression.

It is important to acknowledge the limitations of our work. Due to the principle of our co-aggregate detection method, it is hard to rule out the coincidental positives (the two targets are just spatially adjacent but not truly aggregated together). In our record, the detection positive rate of co-aggregate out of α-syn aggregate signal was not stable. Patient samples tend to exhibit higher positive/co-aggregation rate, however, the distribution of which was haphazard, especially in serum samples **(Figure S7A-D)**. It also demonstrated L1EV as the source analyte has better value than serum free floating ones regarding this issue. On the other hand, we hypothesized an α-syn-amylin co-aggregation but there might be other factors involved, more comprehensive research is needed. Especially, our result in the binding affinity part was not quite consistent to other lab's result (almost in an order of magnitude less) 46. We deduce that it is because we adapted a more classical, conservative technology. Moreover, the size of the aggregates is of vital importance to their toxicity. Namely, oligomers are more toxic than bigger chunks, how the size distribution affected by co-aggregate might be a key area to study. The membrane permeability of the aggregate species is also an aspect to establish its malignancy, which requires more effort.

Above all, our work provides strong support that co-aggregates exist in the circulation system of PD patients and have the potential to serve as a biomarker for diagnostic and stratification. We established a novel detection method against co-aggregates. These results not only provided better biomarker choices but also updated our perception of neurodegenerative diseases.

## Material and Methods

### Preparation of artificial co-aggregates

Co-aggregate product of α-syn-amylin was prepared following a previously reported method 44. In short, amylin peptide (Y-0158, Bioss, China) and α-synuclein monomer (PKSH033771, Elabscience, China) were dissolved in Tris-Buffered Saline respectively, and both adjust to 10μM concentration. Mix 0.5 ml of α-syn monomer solution into 0.5ml freshly prepared amylin solution, in a siliconized microcentrifuge tube (T_70102-681-320, Fisherbrand, USA) 63. Then, a glass bead was added into the tube for agitation and shake in 800rpm/ 37℃ on an oscillating metal bath for 90min. The product was subsequently centrifuged for 20 min at 15000 rpm and collected supernatant. The collected supernatant was then filtered by a 50k ultrafiltration tube (UFC805024, Merck Millipore, USA) to remove excessive monomers.

### Surface-based fluorescence distribution method

#### Immunosorbent setting

We adapt Ultra-TC treated 384 well imaging plate (WP384-4BCCSH, QINGDAO AMA Co. LTD., China) as the carrier of immunoprecipitation, the thickness of its transparent bottom is less than 0.2 mm. The aggregates were captured and identified via “same epitope” principle previously reported 64. In immobilizing the capture antibody, a rapid coating method was implemented 65. The capture antibody of α-syn (sc-12767, Santa Cruz biotechnology, USA) was diluted to 1μg/ml by PBS, then mix in 1% (v/v) of APTES. The mixture was swiftly dripped right against the center of each well using a multisteper, then incubate the plate for 30 min in room temperature. Each step from now on is followed by a twice rinsing by PBST and once by PBS. After coating, blockage of non-specific binding site was implemented by 1% BSA solution for 1 hour in room temperature.

#### Immunosorbent experiment

Based on the carrier abovementioned, we conducted 2 kinds of assays against 2 kinds of samples: 100-fold diluted serum or the lysate of L1EV isolated from the serum. Either way, we add 20μl of the analyte into the readied well of the plate and incubate under 37℃ for 90min. However, plate for the EV lysate was rinsed by PBS instead of PBST.

The fluorescence antibody was based on the most common-used detection antibody of the protein aggregate of α-syn (sc-12767, Santa Cruz biotechnology, USA) or amylin (sc-377530, Santa Cruz biotechnology, USA). The two antibodies are labeled with R-PE (sc-12767PE) and Alexa Fluor 647 (sc-377530 AF647) respectively, both labelled versions are provided by the manufacture. The labelled antibody was 1000-fold diluted in PBS to make a working stock. The working stock was applied into the well by 50μl/well by a multisteper, and incubate in room temperature for 90min, shed from light. After incubation, the well was rinsed and ready for image sampling.

#### Image sampling (fluorescence microscopy)

We conducted fluorescent distribution signal readout on two different platforms, with equivalent configurations.

The readout of PD/HC cohort was achieved by a universal microscopic imaging platform (BZ-X800, Keyence, Japan) mounted with TRITC and Cy7 filter cubes. The above-mentioned immunosorbent plate was mounted on the stage, firstly use 20x lens to locate the bottom surface of the well in bright field and pinpoints the sampling sites (random sampling) within the region of interest (ROI). Then maintain position on the X-Y plane, switch the lens to oil immersion Plan Apochromat 60x/1.40. relocate the Z position of the ROI. Designate an auto-sampling program to scan over 0.5x0.5mm's area in total, which makes up 3% of the ROI. Once the sampling site is configured, a movement sequence is set like “TRITC auto focus, imaging, Cy7 imaging, move to next spot”. Exposure time of TRITC channel was 0.5s while Cy7 was 2s since there's a difference in both target abundance and fluorescence intensity of the dye. Export the two separate pieces of image with correlation noted.

The readout of MSA/PD/HC cohort was achieved by Leica thunder image platform DMi8 (Leica, Germany), on lens of ∞/0.17/OFN24/E HC PL FLUOTAR 63X/1.30 OIL. The rest of setting were the same as above, scale up and down when data processing.

### Transmitted electron microscopy (TEM)

The co-aggregates, soul amylin aggregates, and soul α-syn aggregates were individually characterized using TEM (HT7800, Hitachi, Japan) according to the recommended protocol. All solvents were filtered through 0.22 µm syringe filters prior to use. The aggregate species were prepared as described above, with the concentration adjusted to approximately 0.1 µg/mL. A 10 µL aliquot of the diluted samples was applied to carbon-coated 400-mesh copper grids. Excess sample was removed using filter paper, and the grids were washed before TEM analysis. The samples were fully dried prior to observation.

### Clinical study design

The cross- sectional study comprises two parts: the first part meant to evaluate the diagnostic potential of a-Syn-amylin co-aggregate in distinguishing PD patients out of the HC. The second part aims to study the level of co-aggregate in different synucleinopathies, which comprised three groups: MSA/PD/HC.

For the first part, the diagnosis was based on standard criteria. Control group participants without any neurodegenerative disorders were also recruited. Samples were acquired from Shantou University hospital Department of Neurology and Neurosurgery. Then kindly provided by Prof. Lai. Demographic information and PD related rating scales were provided simultaneously. We included 21 participants in each condition.

For the second part, the diagnosis was based on standard criteria. Each condition (PD, MSA, and HC) included 8 participants. Samples were acquired from the department of rehabilitation of the second affiliated hospital of CUHK-shenzhen (People's hospital of Longgang district) and above-mentioned Shantou University hospital.

Serum samples were obtained by allowing blood samples to clot after collection, followed by 3000g centrifugation for 10min to separate the serum. After collect the serum, infuse 1ml of serum in a sterilized 1.5ml centrifuge tube and centrifuge on 10000g for 15min to clear out cell debris. The cleansed serum samples were preserved in -80℃ freezer in aliquot, thaw on the ice before use. Once an aliquot of serum sample was thawed, it will not be stored again.

### Isolation of brain derived EVs (L1EVs)

Brain derived EV isolation was performed by previously reported method 33. In short, L1CAM antibodies were conjugated onto the magnetic beads, to prepare immunomagnetic beads. Then, the immunomagnetic beads were mixed in 100-fold diluted serum of participant, immunocapture was conducted under ambient temperature for 30 minutes. The magnetic isolated beads were washed three times before use. Add 30μl of prepared lysis buffer to the beads and incubate for 10min. The constituent of lysis buffer can be found in Supp.1. After lysis, gently spin down the mixture and take 20μl of supernatant for analyte.

### Validation of surface-based fluorescence distribution detection system

We adapted commercialized α-syn aggregate (ab218819. Abcam, USA), and homemade co-aggregate as standards. The co-aggregate product stock was adjusted to 1mg/ml concentration (the same as α-Syn aggregate, measured by BCA method). To develop a standard curve, the standards were gradient diluted, start with 1μg/ml, then perform an 8-step dilution with 5-fold between each step. Add 20μl of diluent of each step to the assay well, finally followed by a blank control, each condition was duplicated. In each real sample batch, a validation batch was appended on the very same plate. Batches were found that the standard curve was poor, or massive amylin channel signal presented in α-syn aggregate validation batch, were considered a failure. Failed assay batches were removed from dataset.

### Biomolecular interaction analysis

The affinity interaction between α-synuclein and amylin was examined by surface plasmon resonance (SPR) detection method. amylin-αSynuclein interactions were investigated using a Polariton S-CLASS SPR system (PolaritonLife, China) with amylin immobilized on gold surfaces of carboxymethylated dextran-coated sensor chips by an amine-coupling procedure, using a 0.01 mol/L 4-(2-hydroxyethyl)-1-piperazineethanesulfonic acid (HEPES) buffer containing 0.15 M NaCl and 0.50% (v/v) Tween-20 (pH = 7.4) as an eluent and a flow rate of 10 μl/min. The stock solution of amylin (X mg/mL) was prepared in PBS. The chip surface was activated by derivatization with a freshly prepared mixture of an aqueous solution of 1-ethyl-3-(3-dimethylaminopropyl) carbodiimide (0.4 mol/L) and N-hydroxysuccinimide (0.1 mol/L) (1:1, v/v). The mixture was injected to the chip flow channels sequentially at a flow rate of 10 μl/min. The protein solution (0.01 mg/mL) in a 0.01 mol/L sodium acetate buffer (pH = 5.5) was injected into flow cell B (flow cell A was used as a blank control). The unoccupied sites were blocked by injection of ethanolamine-HCl (1 mol/L, pH = 8.5) for 7 min at a flow rate of 10 μl/min. Both the activation and immobilization procedure were performed at 25 °C.

### Image analysis algorithms

A tailored script for dual-channel image analysis is coded. The program is designed to process (enhance, filter, and organize), extract features, and compare in the two sets of images captured from the two channels. It aims to determine the number of overlapping circular structures and visualize the processing steps and results. Based on OpenCV, the program performs histogram equalization for image enhancement. It defines contour detection and Hough circle detection functions to extract relevant features (each light particle representing an aggregate) and outlines their contours from the enhanced images. The program iterates through images captured from the red (corresponding to α-syn) and blue (corresponding to amylin) channels, calculates the number of overlapping circular structures for each pair of images, and outputs the dataset.

### Statistical analysis

For two-group comparisons (PD vs. HC, Shantou cohort), we performed student t-test using OriginLab 2024 (San Diego, USA). Relationships between co-aggregates and diabetic biomarker HbA1c percentage and PD indicative UPDRS motor scores were analysed with bivariate correlation using Spearman's correlation coefficients. Data from these groups were analysed using receiver operating characteristics. To assess the distribution of the co-aggregate in separating α-synucleinopathies (MSA/PD/HC, Longgang cohort), we compared their count in L1EV and serum by one-way ANOVA. Values with *p* < 0.05 were regarded as significant.

### Cell experiments

The M17 cell line, obtained from Shanghai Institute of Biochemistry and Cell Biology (China), was maintained in DMEM medium (Gibco, C11995500, USA) supplemented with 10% heat-inactivated fetal bovine serum (TransGen Biotech, 301-02, China), under a humidified atmosphere of 5% CO2 at 37°C. 1cm-coverslip was coated with 0.01% poly-L-Iysine for 1 hour at room temperature, washed with PBS. Then seed trypsinized cell in 10000 per well. Cells were cultured for 2 days to reach to ~80% confluency on the coverslip. The cells were exposed with 10μg of amylin (Y-0158, bioss, China) or α-syn aggregates (ab218819, abcam, USA) for three hours. Amylin and α-syn aggregates were provided in 1mg/ml of PBS. Blank were used as control.

For protein analysis: Harvest the cell after the expose by lysis buffer, containing: 50 mM Tris-HCl, 150 mM NaCl, 1 mM EDTA, 1% Triton X-100, 0.1% SDS, 0.5% sodium deoxycholate, pH 7.4 and complete protease inhibitor mixture cocktail (SigmaAldrich). Lysis on ice for 10 min, and ready for the Co-immunoprecipitation.

For immunocytochemistry: After the expose, immobilize the cell with 1% Paraformaldehyde (P1111, solarbio, China) for 10min, rinse three times by PBS. Prepare fluorescent antibody against α-syn and amylin mixture solution, dilute to both 1000-fold by PBS, then soak the coverslip in copious mixture solution for 1hour in room temperature. Rinse for three times and mount the coverslip onto the slide using gelatin mounting medium.

### Co-immunoprecipitation (co-IP)

Two kinds of co-IP were performed. The first one was performed by cell lysate mentioned above, the other one was performed by artificial co-aggregate product.

The cell lysates were incubated with the SC211 antibody (1:100) for 1 h at RT. Then, 20μl of Protein A/G beads (Beaver Biotechnology) was added and incubated for 1 h at RT on a rotator. The immunoprecipitates were collected by magnet and washed 3 times with TBS buffer. The samples were boiled in SDS loading buffer for 5 min and subjected to Western blot analysis using antibodies against α-syn and amylin. For co-IP performed by artificial co-aggregate product, the incubation time was shortened to 0.5h.

### Immunogold labeling

Prepared artificial co-aggregates were subjected to immunogold labeling to validate its composition. Samples were applied to carbon-coated EM grids and sequentially incubated with primary antibodies targeting α-syn (ab209538) and amylin (sc-377530). Corresponding secondary antibodies conjugated with colloidal gold particles of different diameters—10 nm for α-syn and 35nm for amylin—were used to enable distinct visualization. Control grids treated only with secondary antibodies served to verify labeling specificity. The samples were negatively stained with uranyl acetate and examined under a transmission electron microscope operating at 100 kV (HT7800, Hitachi, Japan).

### MALDI-TOF mass spectrometry analysis

To confirm that α-synuclein (α-syn) and amylin were in their monomeric forms, Matrix-Assisted Laser Desorption/Ionization Time-of-Flight (MALDI-TOF) mass spectrometry was performed using a Bruker Autoflex Max mass spectrometer (Bruker Daltonics, Bremen, Germany). Stock solutions of α-syn and amylin (20 μM each) were prepared in Tris-buffered saline (TBS, pH 7.4). Samples were diluted 1:10 in a matrix solution of sinapinic acid (10 mg/mL in 50% acetonitrile, 0.1% trifluoroacetic acid). A 1 μL aliquot of the sample-matrix mixture was spotted onto a MALDI target plate and air-dried. Spectra were acquired in positive ion linear mode, with a mass range of 5-20 kDa, and calibrated using protein standards. Monomeric forms of α-syn and amylin were identified by their expected molecular weights.

### *In-silico* methods

#### Preliminary structure prediction

The alphafold-3 online version was used to generate preliminary molecular structure. (https://alphafoldserver.com/) On the server page, entry type was selected as “protein”, the full-length sequence of α-syn and amylin, were paste respectively. Copy numbers were designated as 1 or 5. File packages containing .cif and .pdb were then downloaded for further analysis.

#### Establishment of peptide models and force field setup

α-syn (104-137) and full-length amylin were selected as the representative fragments to investigate the a-syn-amylin coaggregation behaviors. α-syn (104-137) had 34 amino acids (N terminus --- APQEGILEDMPVDPDNEAYEMPSEEGYQDYEPEA --- C terminus), while amylin had 37 amino acids (N terminus --- KCNTATCATQRLANFLVHSSNNFGAILSSTNVGSNTY---C terminus). The peptide atomistic models were set up at pH 7.4, corresponding to that of the human body environment and the experimental setup. 10 Na^+^ ions were added to neutralize the peptide system. Overall, the simulation cell contained 2 peptides, 11749 water molecules, 10 Na^+^ ions, and 33 Na^+^Cl^-^ ion pairs to keep the Na^+^ ion concentration of 0.15 M. Typically, a 3D periodic box with size of 130.000×50.000×60.000 Å^3^ was utilized to study binding and coaggregation of peptides. We utilized CHARMM36 and INTERFACE force field (IFF) for peptide molecules and ions, respectively 66,67. The TIP3P water models were utilized as the solvent in this work.

#### Molecular dynamics simulation protocol

MD simulations were carried out using the Nanoscale Molecular Dynamics program (NAMD) program 68. The whole system was first minimized for 100 steps to minimize the geometry and energy. Then, the system was relaxed in the isothermal-isobaric ensemble (NPT) condition for 0.5 ns with a timestep of 2.0 fs under the pressure of 101.3 kPa. After that, the canonical ensemble (NVT) was used to equilibrate the system for 20 ns to study the structural and energy information of peptides. All atoms were allowed to move freely during the simulation. The temperature was controlled at 298.15 K using the Langevin thermostat with a damping coefficient of 1 ps^-1^. A spherical cutoff of 12 Å was applied for the summation of pair-wise Lennard-Jones interactions and electrostatic (Coulomb) interactions. The summation of the electrostatic interactions (Coulomb) was completed using the Particle Mesh Ewald (PME) method with accuracy of 10^-6^. Specifically, rigid bonds were employed for TIP3P water models. Simulations were repeated 3 times to obtain average results. After the simulations, Visual Molecular Dynamics (VMD) and self-developed python scripts were used to analyze the simulation results 69.

## Supplementary Material

Supplementary methods, figures and tables.
