# Supplementary material for: Unraveling α-synuclein and amylin co-aggregation: pathological insights and biomarker development for Parkinson's disease
Source: Theranostics. 2025 Jun 20;15(15):7409–24. doi: 10.7150/thno.112396 (PMC12315819; doi:10.7150/thno.112396)
Supplement: Supplementary file 1 — Supplementary methods, figures and tables. [file thnov15p7409s1.pdf]

Supplementary 1

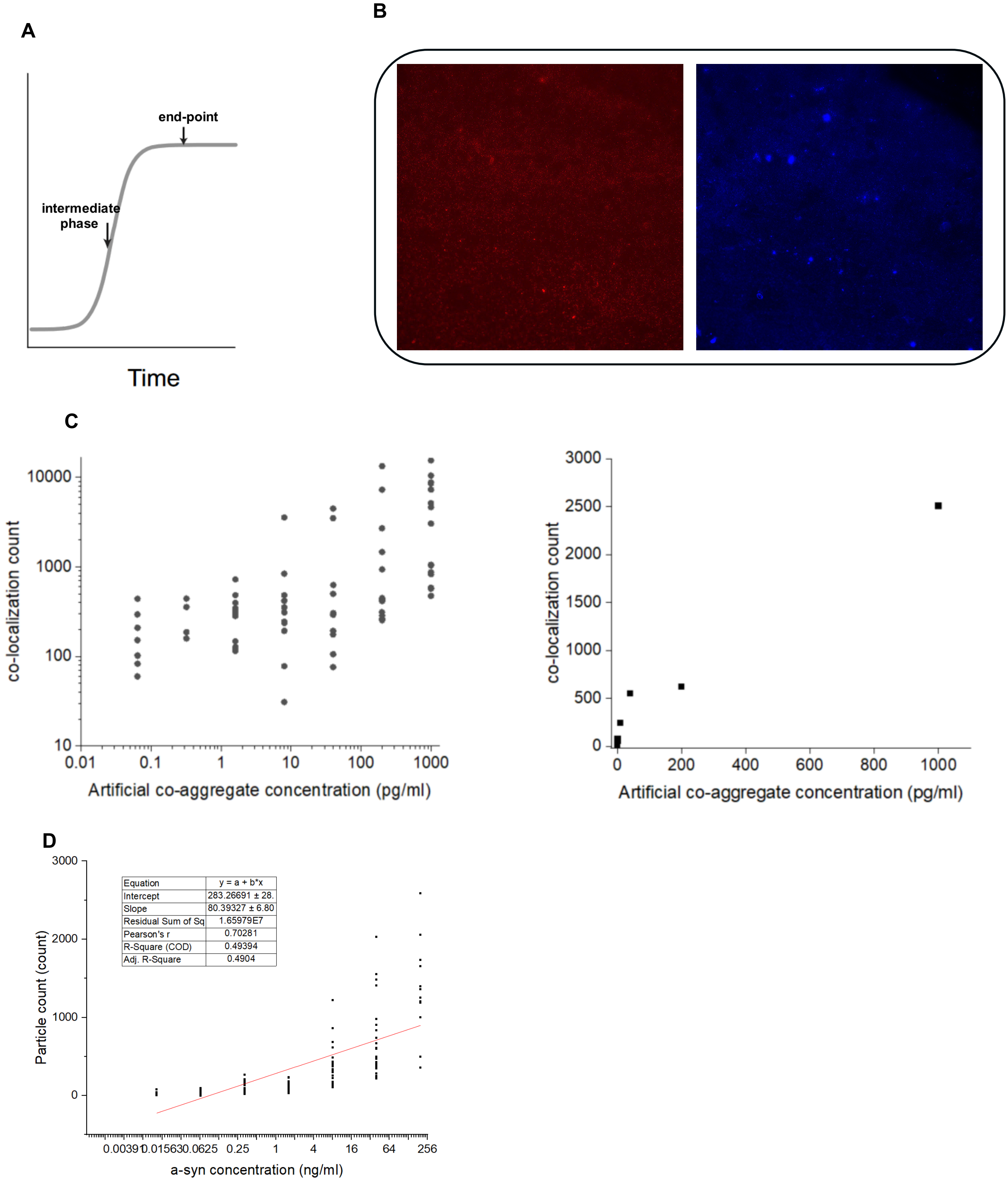

Supplementary Figure 1. Establishment of surface fluorescent distribution measurement that using artificial co-aggregates for pilot. A, schematic diagram showing the timepoint that we harvest artificial co-aggregate. **B,** Representative image showing the raw picture from the two channels (showing high concentration artificial co-aggregate). **C,** Left and right, showing the detection count positively correlate to artificial co-aggregate concentration. **D,** System performance test by α-syn aggregates.

Supplementary 2

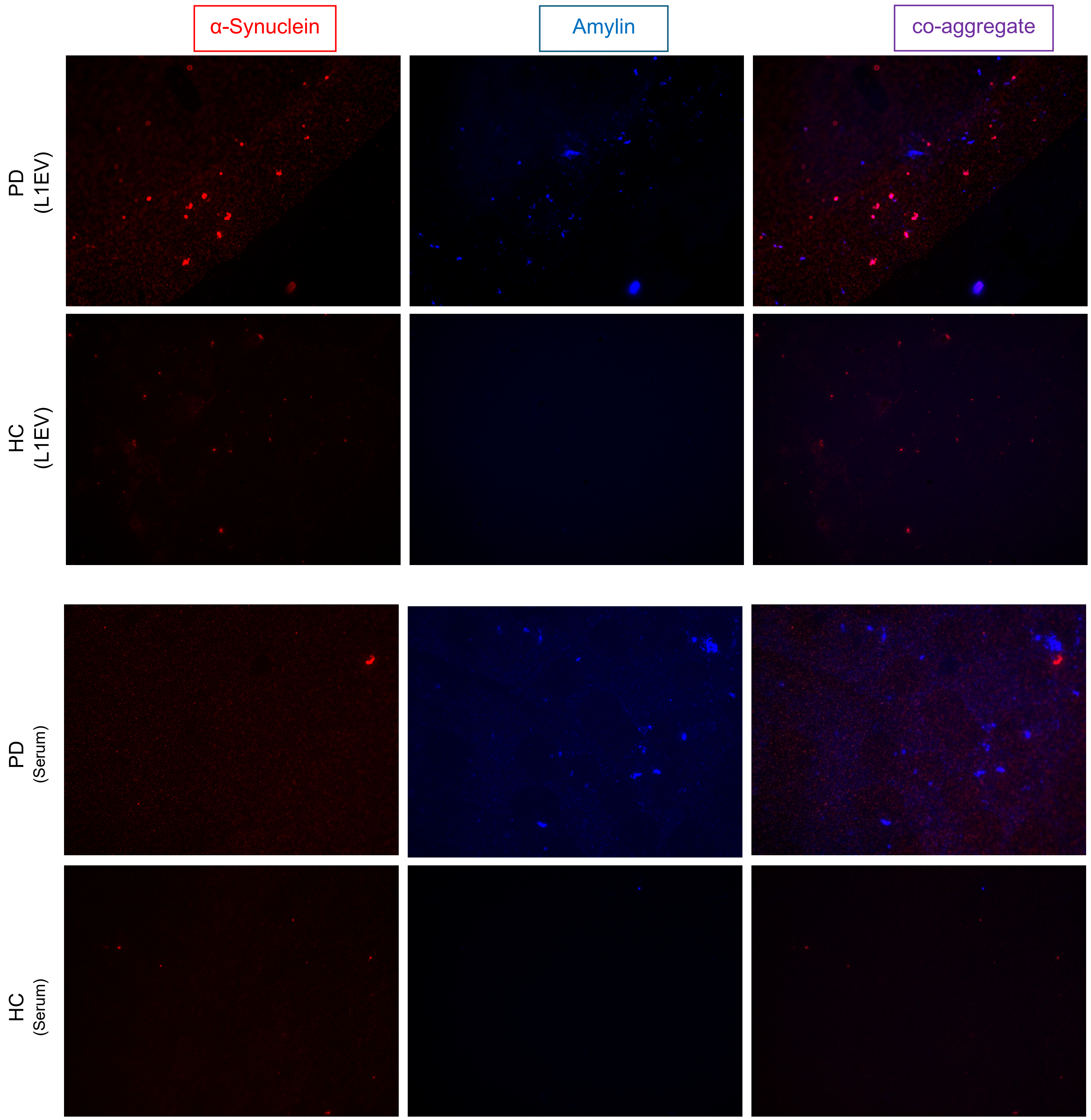

Supplementary Figure 2. Panel to PD vs HC microscopic sampling image in two channels and merged image (representative). Up to down, L1EV sample results from PD patients, L1EV sample results from health individuals, serum (free floating) sample results from PD patients, serum (free floating) sample results from health individuals.

Supplementary 3

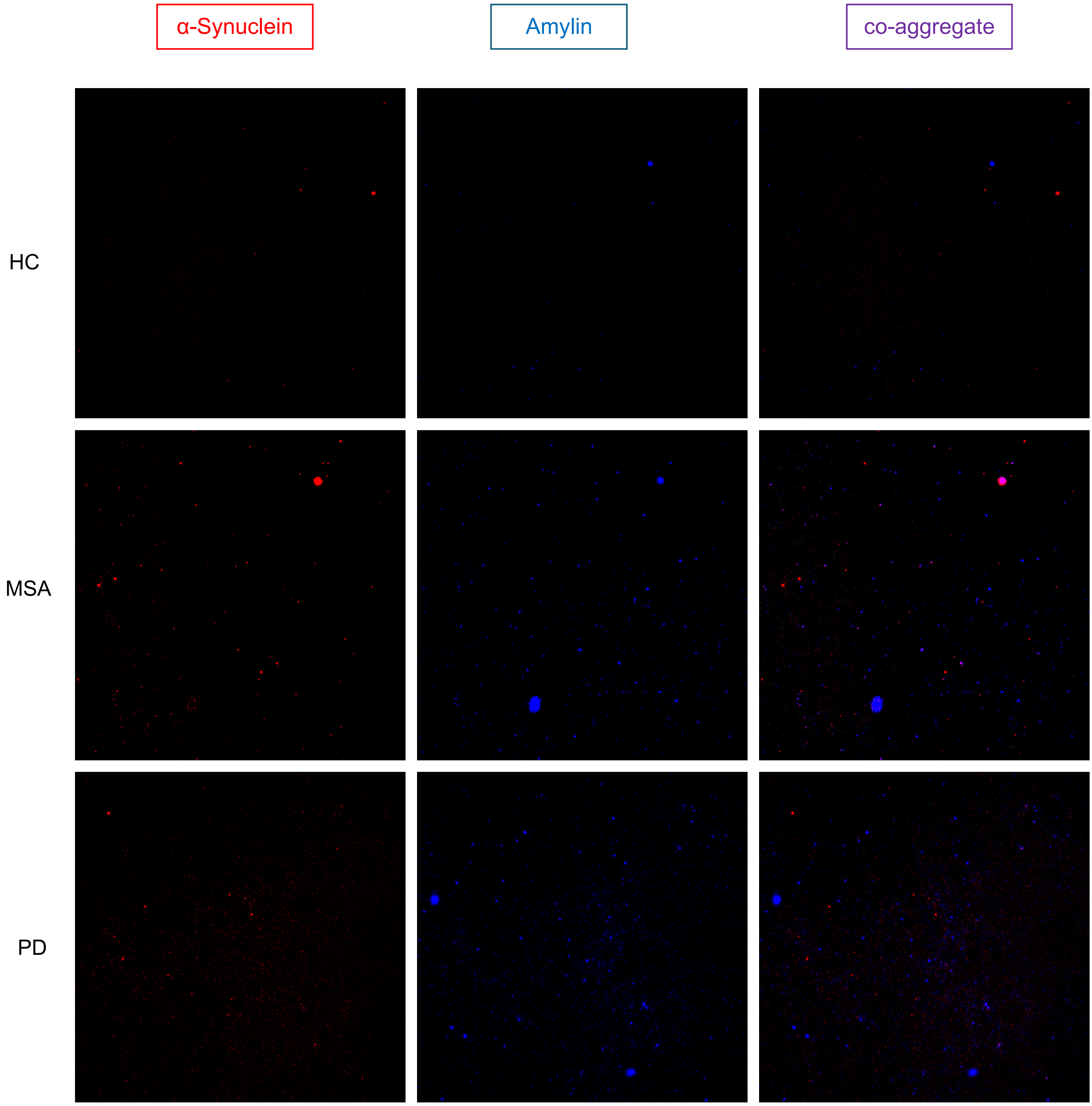

Supplementary Figure 3. Panel to PD vs HC microscopic sampling image in two channels and merged image. Up to down, L1EV sample results from health individuals, L1EV sample results from MSA patients, L1EV sample results from PD patients.

Supplementary 4

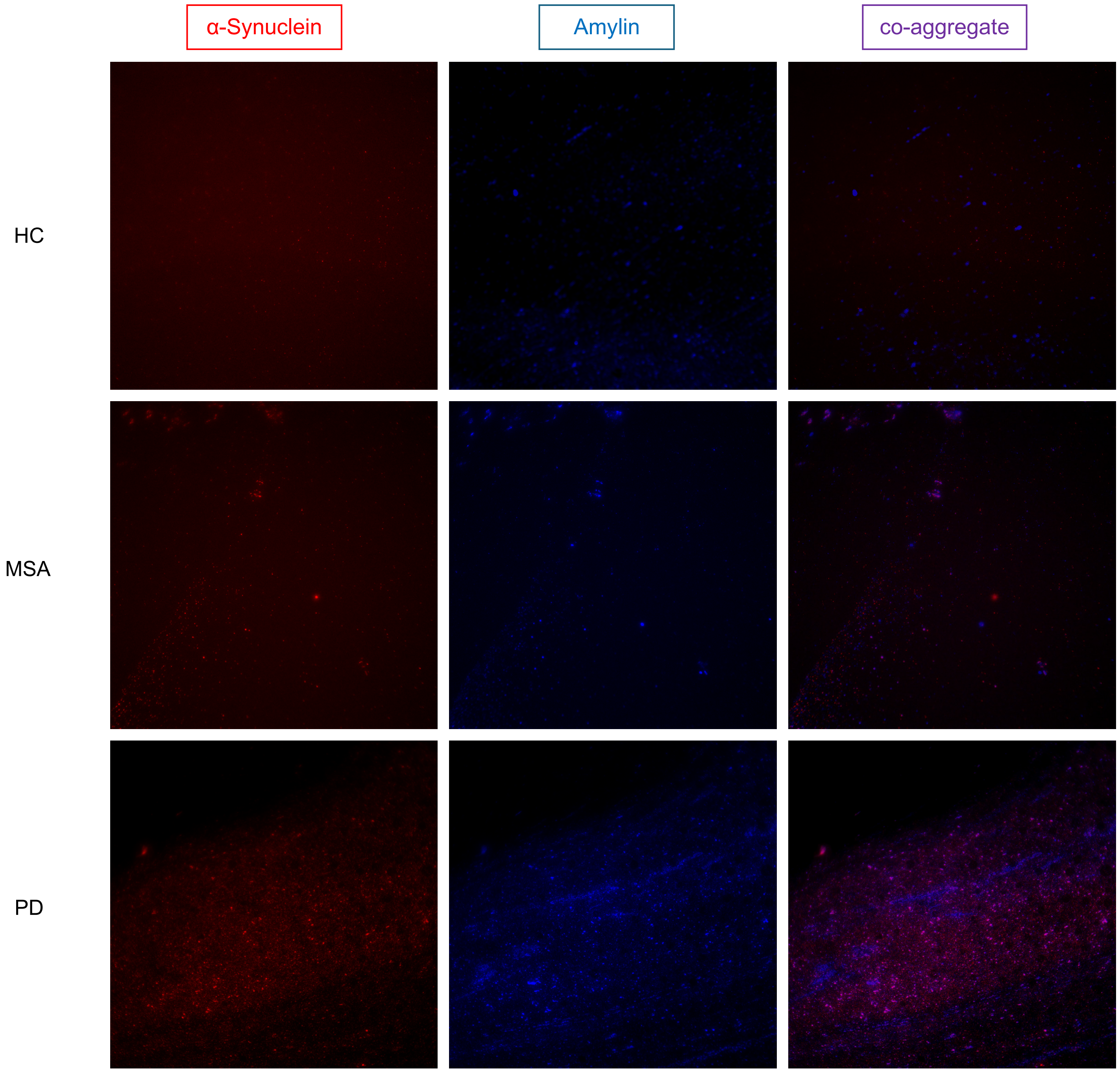

Supplementary Figure 4. Panel to PD vs HC microscopic sampling image in two channels and merged image. Up to down, serum (free floating) sample results from health individuals, serum (free floating) sample results from MSA patients, serum (free floating) sample results from PD patients.

Supplementary 5

Table 1. Demographic and clinical information on patients and controls that donated serum samples.

	PD	HC	p (PD vs. HC)
No. of individuals	21	21	N/A
Female [number (percentage)]	14(66.7)	8(37)	N/A
Age [years ± SD]	62.3 ± 10.7	50.8 ± 11.1	n.s.
Disease duration [years ± SD]	4.3 ± 3.4	N/A	N/A
MDS-UPDRS III [score ± SD]	44.8 ± 11.1	N/A	N/A
MoCA [score ± SD]	24.2 ± 4.2	N/A	N/A
Hoehn and Yahr [score ± SD]	2.6 ± 0.5	N/A	N/A

Abbreviations: PD, Parkinson disease; HC, healthy controls; MoCA, Montreal Cognitive Assessment; NA, not applicable; MDS-UPDRS III Movement Disorder Society's Unified Parkinson's Disease Rating Scale Part III; n.s. not significant.

Table 2. Demographic and clinical information on patients and controls that L1EVs were isolaed from serum samples.

	PD	HC	p (PD vs. HC)
No. of individuals	20	20	N/A
Female [number (percentage)]	12(60)	7(35)	N/A
Age [years ± SD]	63.2 ± 10.7	50.8 ± 11.1	n.s.
Disease duration [years ± SD]	4.3 ± 3.4	N/A	N/A
MDS-UPDRS III [score ± SD]	42.4 ± 8.5	N/A	N/A
MoCA [score ± SD]	24.5 ± 4.4	N/A	N/A
Hoehn and Yahr [score ± SD]	2.6 ± 0.5	N/A	N/A

Abbreviations: L1EV, L1CAM positive extracellular vesicles; PD, Parkinson disease; HC, healthy controls; MoCA, Montreal Cognitive Assessment; NA, not applicable; MDS-UPDRS III Movement Disorder Society's Unified Parkinson's Disease Rating Scale Part III; n.s. not significant.

Supplementary Tables. Tables showing demographic information correspond to HC vs PD co-aggregate measurements. Table 1 represents free floating serum-based results. Table 2 represents L1EV samples.

Supplementary 6

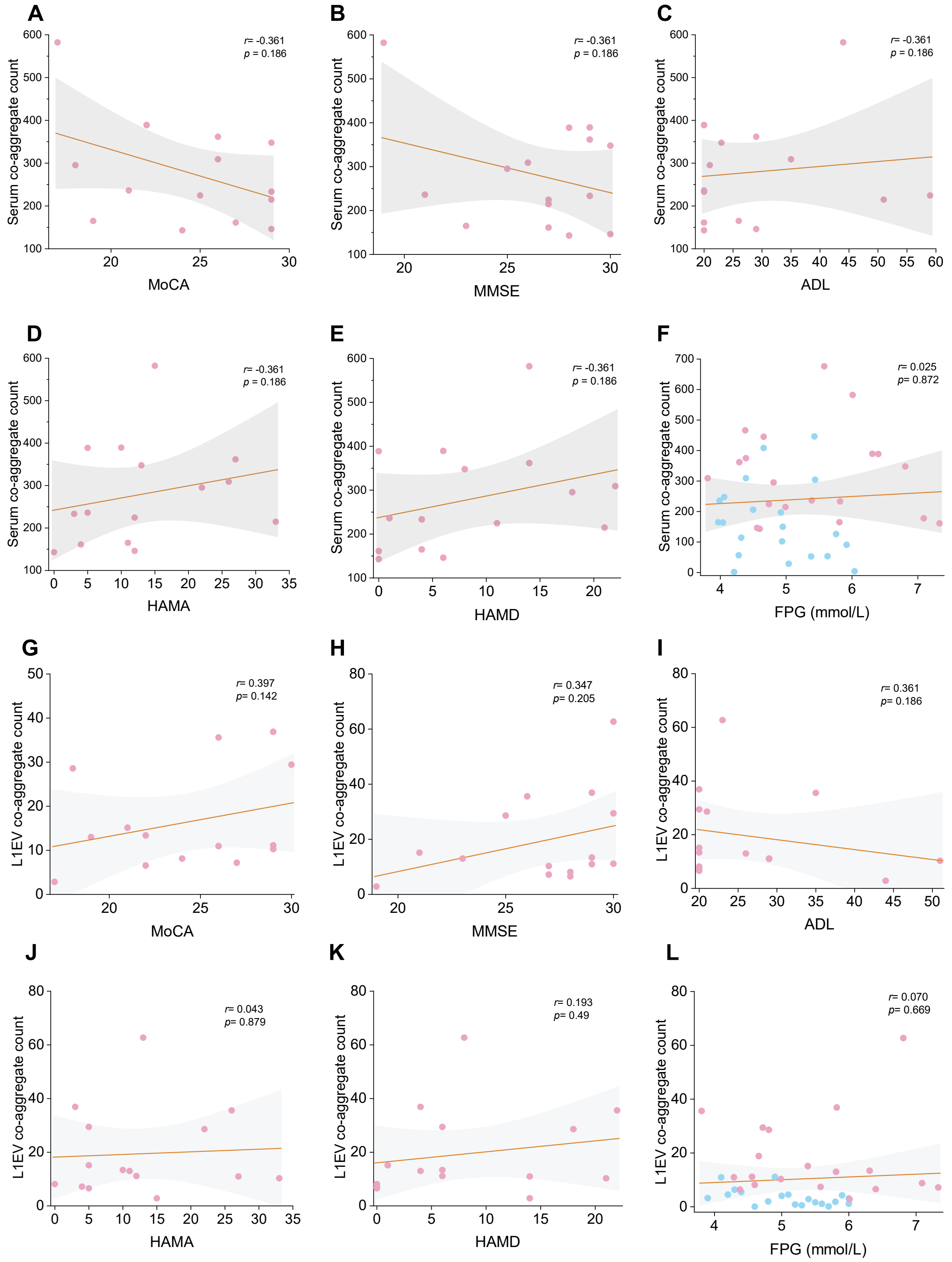

Supplementary Figure 5. Correlation analysis regarding non-motor scales of PD with co-aggregate counts. A-F, serum free floating co-aggregate count. G-L, L1EV carried co-aggregate count. MoCA, Montreal Cognitive Assessment. MMSE, Mini-Mental State Examination. ADL, Activities of Daily Living. HAMA, Hamilton Anxiety Rating Scale, HAMD, Hamilton Depression Rating Scale. FPG, Fasting Plasma Glucose.

Supplementary 7

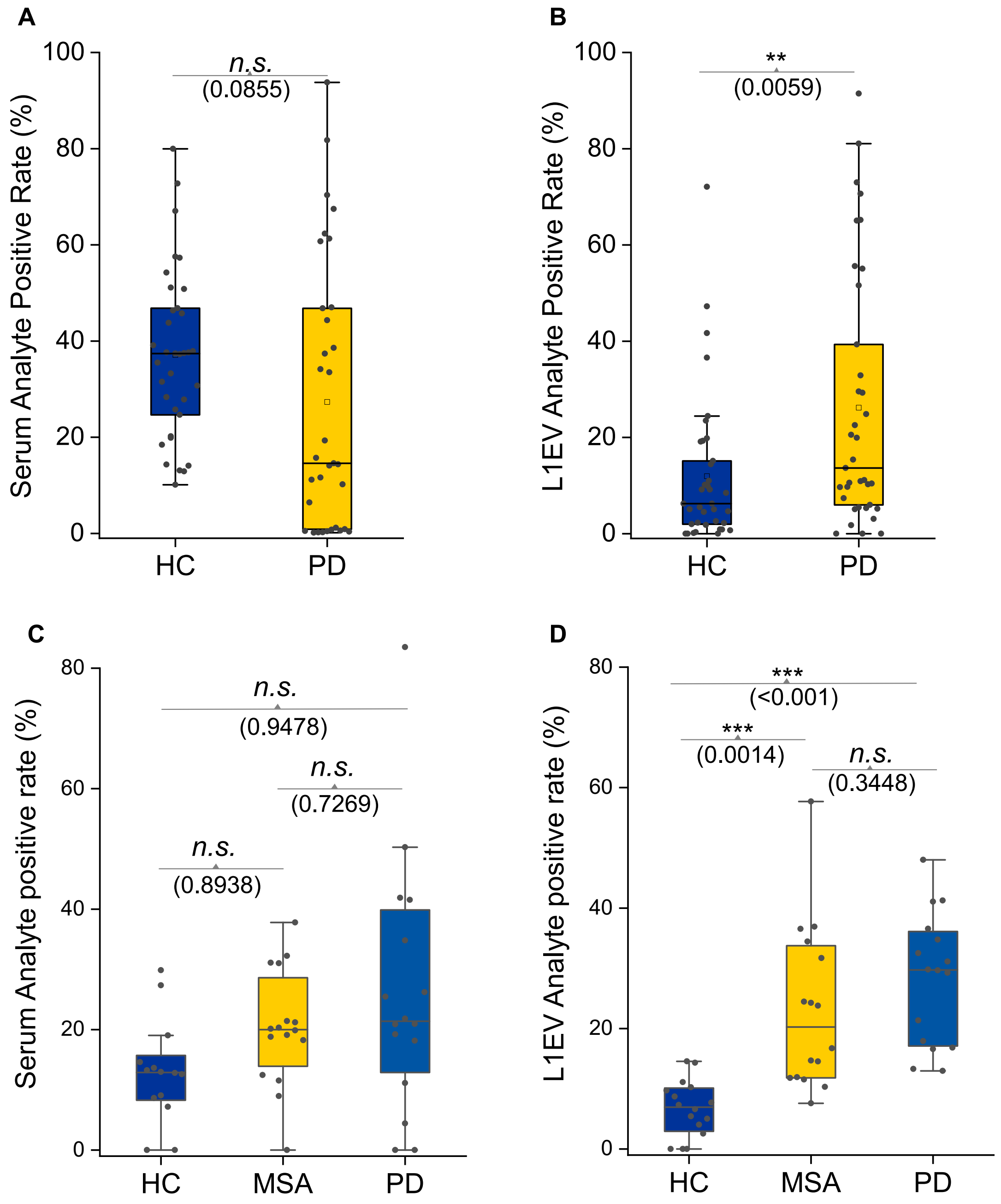

Supplementary Figure 7. Positive rate of co-localization in each analysis. A, serum free floating co-aggregate (colocalization positive rate) ratio in PD-HC cohort. B, L1EV co-aggregate (colocalization positive rate) ratio in PD-HC cohort. C, serum free floating co-aggregate (colocalization positive rate) ratio in PD-MSA-HC cohort. D, L1EV co-aggregate (colocalization positive rate) ratio in PD-MSA-HC cohort.

Supplementary 8

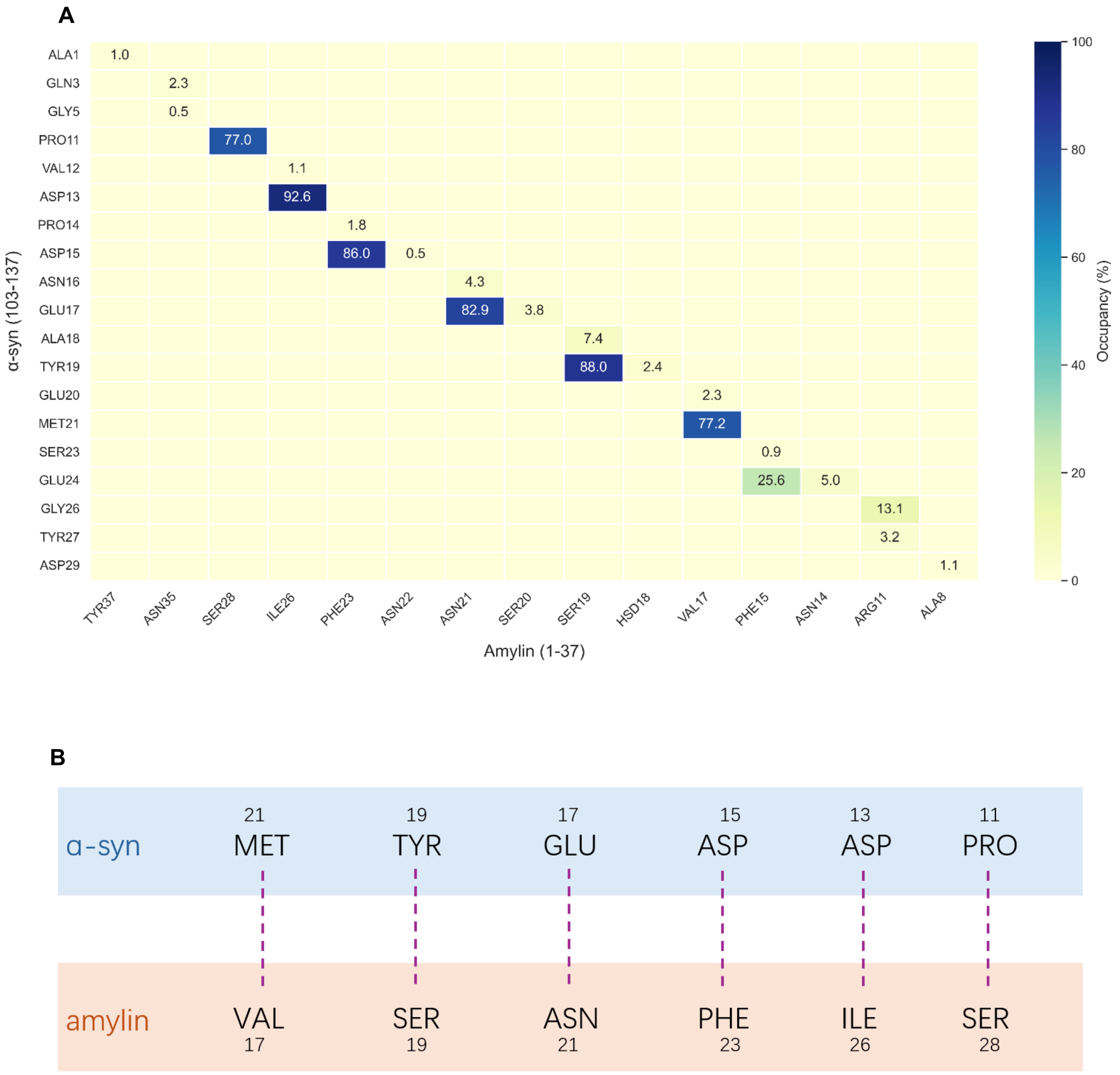

Supplementary Figure 8. Molecular dynamics simulation on corresponding residue pairs. A, Hydrogen bond occupancy heatmap. B, Diagram showing amino acid residue pairs on the two chains that are likely to form hydrogen bonds.

Supplementary 9

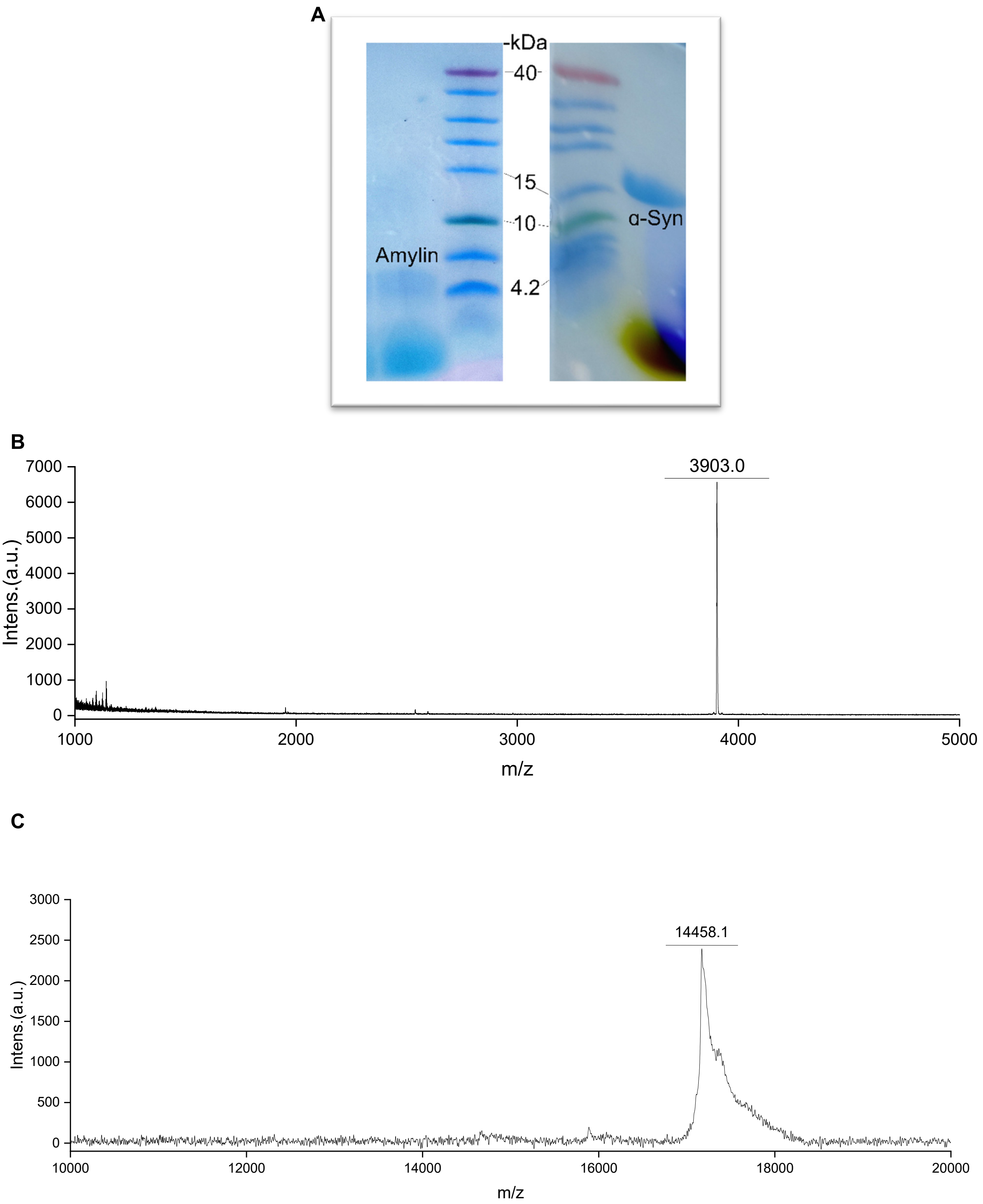

Supplementary Figure 9. Co-aggregate partner's validation. A, Native-PAGE result of α-syn and amylin stock solution before the initiation of the artificial co-aggregation experiment. B, MALDI-TOF analysis result of amylin used prior to the artificial co-aggregation experiment, showing monomeric state. C, MALDI-TOF analysis result of α-syn used prior to the artificial co-aggregation experiment, showing monomeric state.

Supplementary 10.1

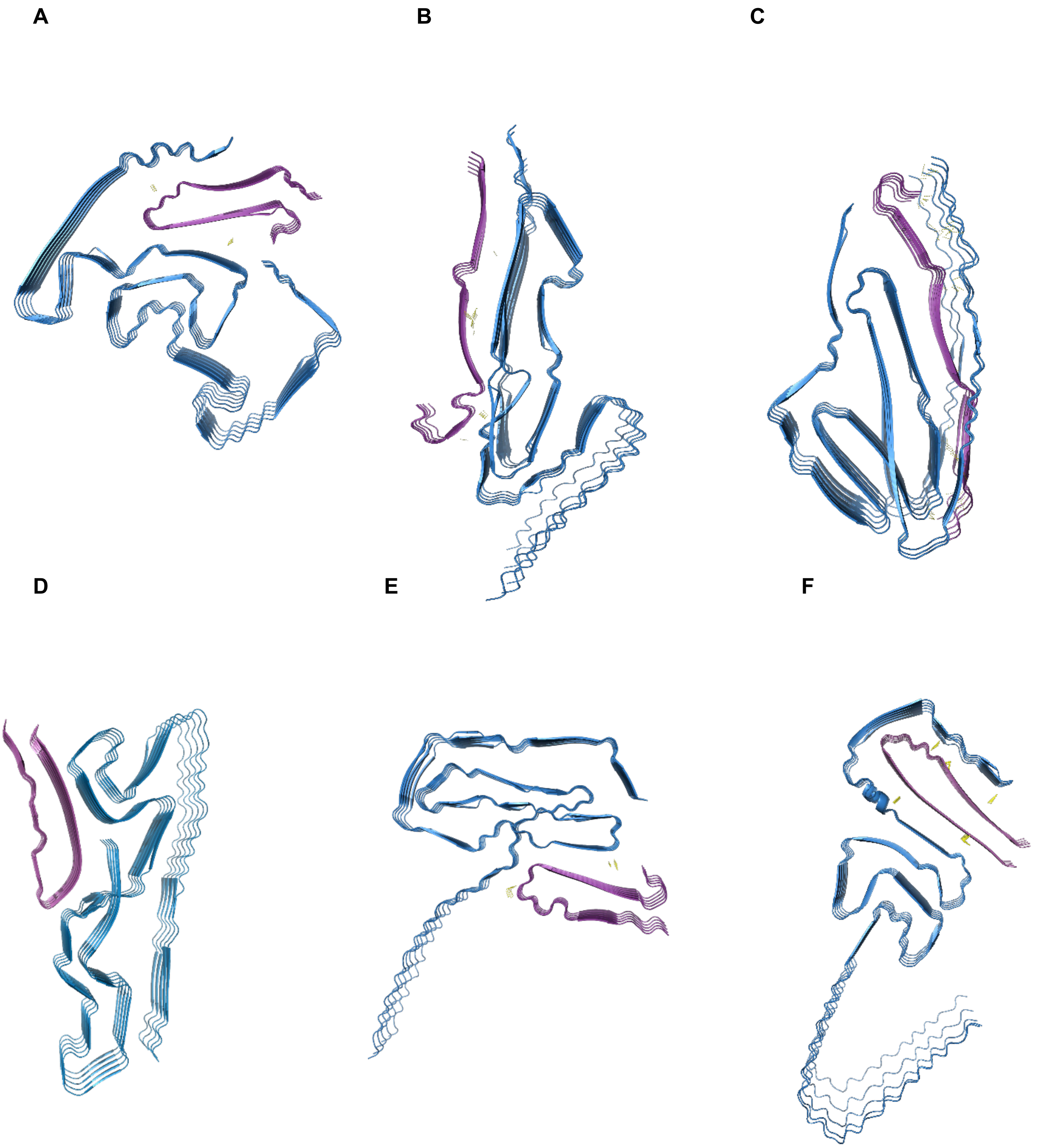

Supplementary Figure 10. Alphafold-3 predicted α-syn and amylin co-aggregate configuration. A-G, representative configurations that comprises 5 α-syn chains and 5 amylin chains. H-L, representative configurations that comprises one α-syn chains and one amylin chain.

Supplementary 10.2

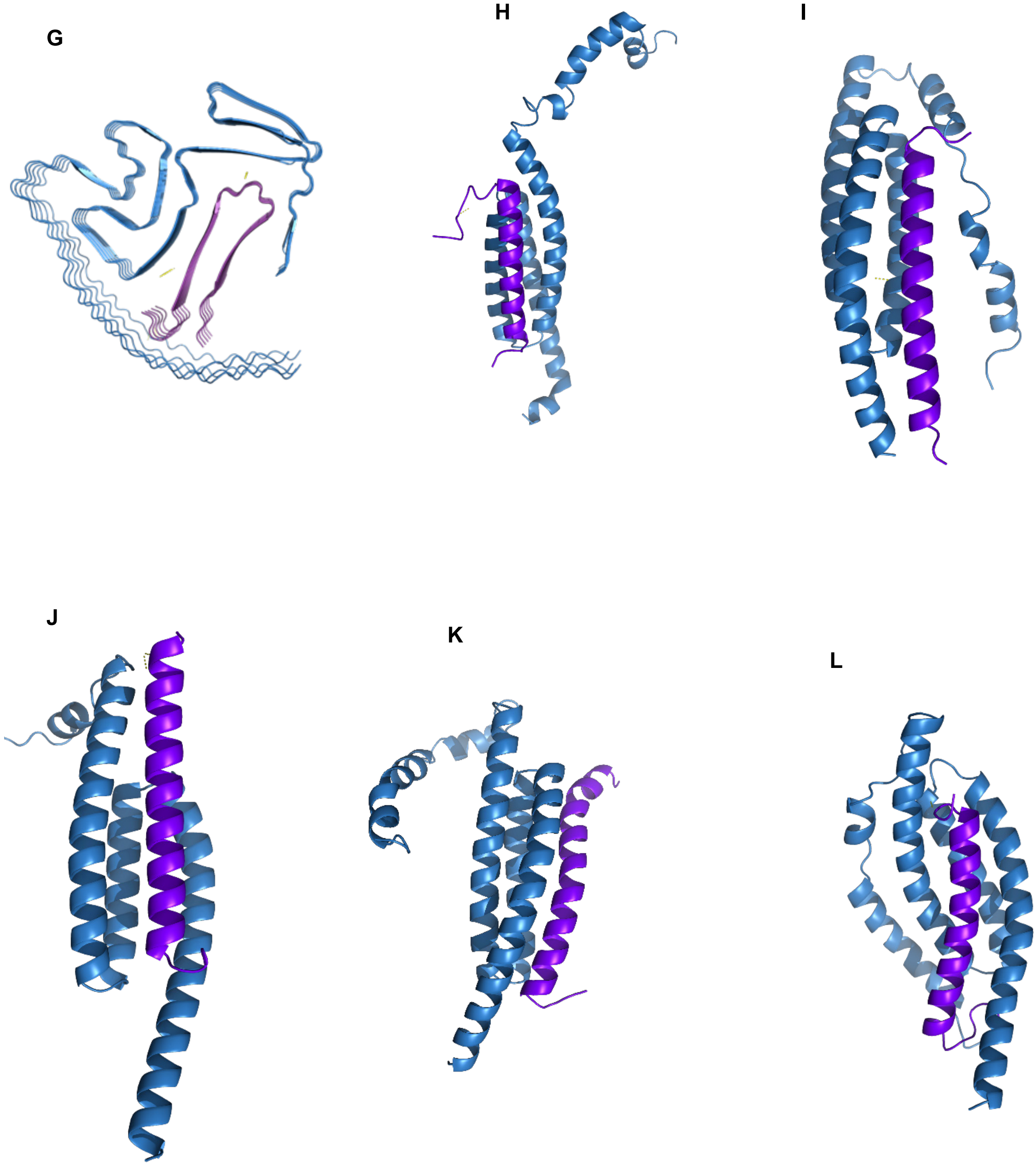

Supplementary 11

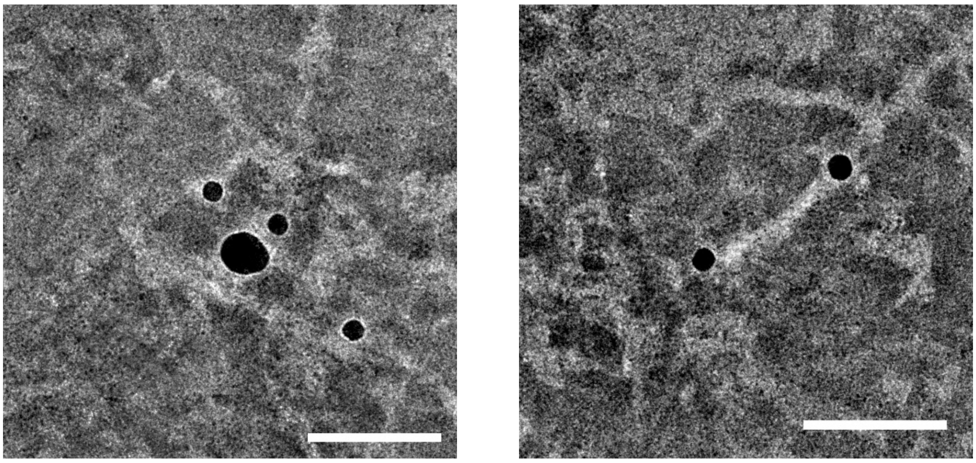

Supplementary Figure 11. Immunogold dual-labeling of co-aggregate *vs* α-syn aggregate. Left, representative image of co-aggregate decorated by both types of nanogold-conjugated secondary antibody. Right, representative image of α-syn aggregate decorated by only one type of nanogold-conjugated secondary antibody. Scale bar = 100nm.
